# Ancestral mitogenome capture of the Southeast Asian banded linsang

**DOI:** 10.1371/journal.pone.0234385

**Published:** 2020-06-30

**Authors:** Johanna L. A. Paijmans, Axel Barlow, Kirstin Henneberger, Joerns Fickel, Michael Hofreiter, Daniel W. G. Foerster

**Affiliations:** 1 Institute for Biochemistry and Biology, University of Potsdam, Potsdam, Germany; 2 Leibniz Institute for Zoo- and Wildlife Research, Berlin, Germany; 3 School of Science and Technology, Nottingham Trent University, Nottingham, United Kingdom; National Cheng Kung University, TAIWAN

## Abstract

Utilising a reconstructed ancestral mitochondrial genome of a clade to design hybridisation capture baits can provide the opportunity for recovering mitochondrial sequences from all its descendent and even sister lineages. This approach is useful for taxa with no extant close relatives, as is often the case for rare or extinct species, and is a viable approach for the analysis of historical museum specimens. Asiatic linsangs (genus *Prionodon*) exemplify this situation, being rare Southeast Asian carnivores for which little molecular data is available. Using ancestral capture we recover partial mitochondrial genome sequences for seven banded linsangs (*P*. *linsang*) from historical specimens, representing the first intraspecific genetic dataset for this species. We additionally assemble a high quality mitogenome for the banded linsang using shotgun sequencing for time-calibrated phylogenetic analysis. This reveals a deep divergence between the two Asiatic linsang species (*P*. *linsang*, *P*. *pardicolor*), with an estimated divergence of ~12 million years (Ma). Although our sample size precludes any robust interpretation of the population structure of the banded linsang, we recover two distinct matrilines with an estimated tMRCA of ~1 Ma. Our results can be used as a basis for further investigation of the Asiatic linsangs, and further demonstrate the utility of ancestral capture for studying divergent taxa without close relatives.

## Introduction

Ancestral hybridisation capture is a method that employs RNA or DNA oligonucleotides (“baits”) based on a reconstructed ancestral sequence of the target region to selectively enrich genetic libraries prior to sequencing, thus increasing target sequence output compared to standard shotgun sequencing. As such, it can be employed in cases where no closely related reference sequence species is available, as has been shown by its successful application to recover a mitochondrial genome from the extinct glyptodont [1]. Hybridisation capture in general is particularly suited to the genetic analysis of ancient and historical specimens, where DNA is likely to be highly degraded and potentially contaminated. Additional case studies on the application of ancestral capture are therefore useful to establish this approach as a standard method for the genetic investigation of species which lack (extant) close relatives.

**Fig 1:**
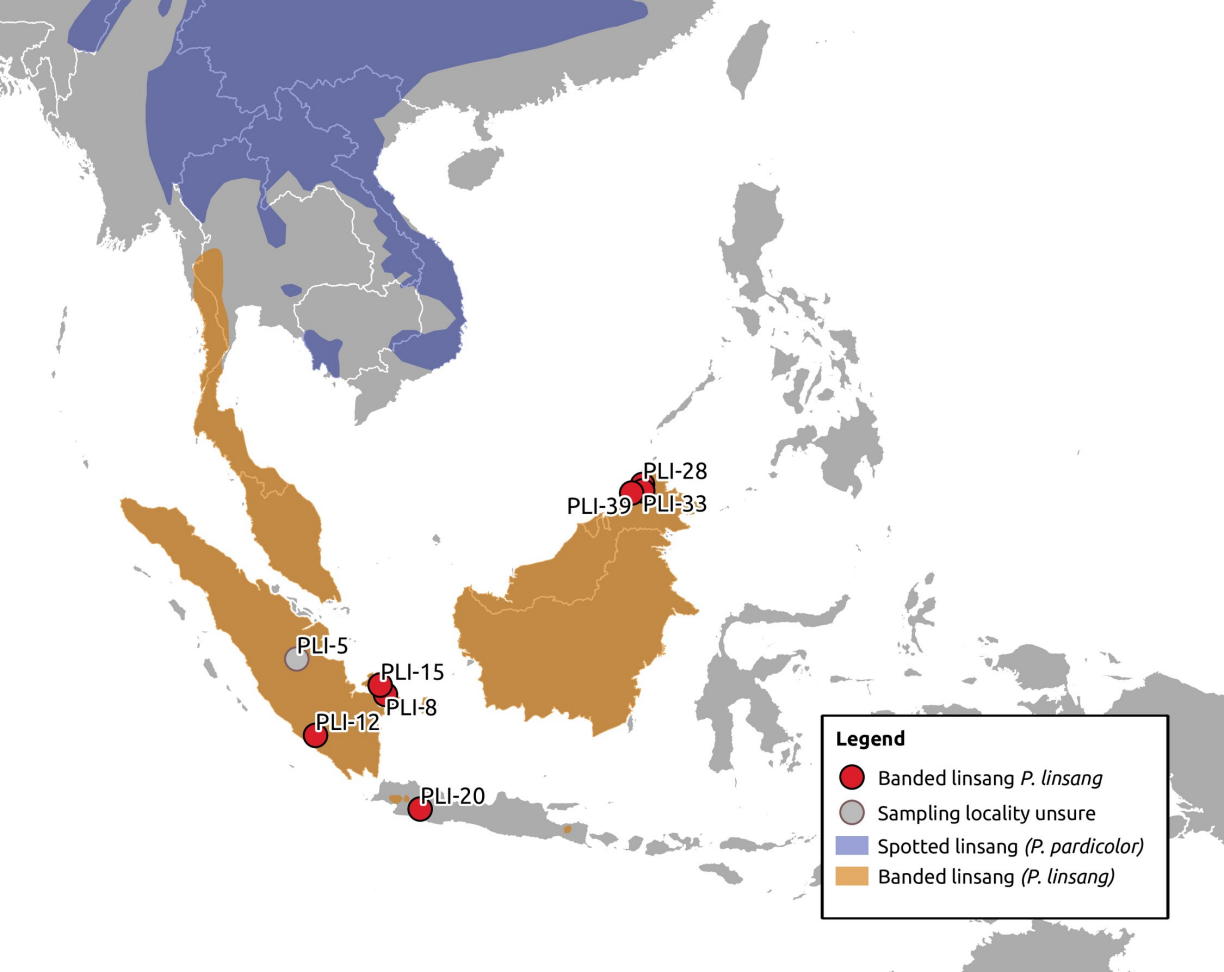
Approximate location of sampling sites. Distribution of the two linsang species (banded linsang P. linsang and spotted linsang P. pardicolor) are indicated in orange and blue, respectively (adapted from [45, 46]).

Asiatic linsangs (*Prionodon* sp.) are an example of a genus for which little molecular data exists, and sampling fresh material is challenging. Linsangs are small, genet-like carnivores with a wide Southeast Asian distribution (Fig 1). The monogeneric family Prionodontidae consists of two extant species: the banded linsang (*Prionodon linsang*) that inhabits Sundaic southeast Asia, and the spotted linsang (*Prionodon pardicolor*) that inhabits much of the non-Sundaic southeast Asian mainland (Fig 1). While these species have been characterized morphologically [2], and some basic knowledge exists regarding their ecology [3–7], little is known about the populations of these elusive nocturnal carnivores. Molecular data has helped to resolve their phylogenetic relationship to other carnivorans—resulting in the establishment of their own family (Prionodontidae), rather than being placed within the Viverridae [8–10]. However, very limited investigation of intraspecific genetic variation has been done for the spotted linsang [11], and equivalent data for the banded linsang is lacking entirely. Furthermore, there is no knowledge on how populations are structured for either linsang species.

In order to assess the intraspecific variation of the banded linsang, we investigated specimens sampled at natural history museums (30–100 years old) from several parts of its Sundaic distribution (Sumatra, Borneo, and Java). We utilised a capture approach based on a reconstructed ancestral mitogenome (henceforth “ancestral capture”) sequence of the Felidae (the closest related family, ca. 28 million years (Ma) divergence from the *Prionodon* lineage; from Paijmans et al. [12]) to enrich the linsang genetic libraries for mitochondrial DNA sequences. The recovered sequences provided several novel insights into the evolution of the Asiatic linsangs. We also evaluated the efficiency of our capture strategy for regions of the mitogenome with high and with low sequence similarity, leading to recommendations for future ancestral capture experiments.

## Materials & methods

### Samples

Epithelial tissue from skulls or skins, or maxillo-turbinal bones, were obtained for ten historical banded linsang samples from natural history museums, eight of which yielded DNA (Table 1) and two failed to yield any mitochondrial DNA (S1 Table of S1 Data). The successful specimens originated from Borneo (*N* = 3), Sumatra (*N* = 2), Bangka Island (N = 2) and Java (*N* = 1). The provenance of one of the two Sumatran samples (PLI-5) has been drawn into question, as the collector of this specimen (M. Bartels) was primarily known for his work in Java, and only rarely visited Sumatra. Therefore, it is possible that this sample could represent an individual from Java instead of Sumatra.

**Table 1:** Sample information and basic mitogenome recovery results. More detailed sequence statistics can be found in S1 Table of S1 Data.

Sample code	Locality	Collection	Collection year	Collector	Collection voucher code	Mitogenome recovery method	No. mapped reads (unique)	Portion of mitogenome recovered at ≥3x sequencing depth	Average Read depth	Genbank Accession number
PLI-5	Sumatra, possibly Java (see text)	Naturalis, Leiden	1954	M. Bartels	14687	Capture	13,984	50.95%	51.0	MT559408
PLI-8	Bangka Island	Naturalis, Leiden	*Unknown*	Sody	33550	Capture	33,015	85.76%	149.8	MT559409
PLI-15	Bangka Island	Naturalis, Leiden	1986	Coll. Maaskamp	34820	Capture	23,842	68.88%	135.0	MT559411
PLI-20	West Java	Naturalis, Leiden	*Unknown*	Beck	34769	Capture	34,277	75.30%	146.2	MT559412
PLI-28	Kinabalu Park, Borneo	Sabah Parks	1988	*Unknown*	18616	Capture	55,243	90.02%	276.0	MT559413
PLI-33	Murug, Ranau, Borneo	Museum Sabah	1976	Taraju Kintarong	27	Capture	12,231	69.27%	59.9	MT559414
PLI-39	Kambu, Penampang, Borneo	Museum Sabah	1971	*Unknown*	392	Capture	42,510	91.63%	220.3	MT559415
PLI-12*	Pagaralam, Sumatra	Naturalis, Leiden	1919	Batenburg	34763	Shotgun	53,797	99.12%	39.1	MT559410

* = sample for which a complete mitochondrial genome was assembled using shotgun sequencing.

### Laboratory procedures

#### DNA extraction & library preparation

All pre-PCR steps were performed in dedicated ancient DNA facilities, with appropriate decontamination procedures in place. Digestion was performed using a non-destructive extraction buffer consisting of 5M of GuSCN, 25 nM NaCl, 50 mM of Tris, 20 mM of EDTA, 1% Tween-20 and 1% ß-mercaptoethanol [13], and binding and washing steps were performed following Dabney et al. [14]. Single-stranded DNA libraries [15,16] and double-stranded DNA libraries [17,18] were prepared from the resulting extracts. For the majority of samples, endogenous content was estimated using low-level shotgun sequencing (appr. 1 million reads per library, 75bp paired- and single-end reads) prior to enrichment. For sample PLI-12, additional shotgun sequencing was performed in order to de-novo assemble a complete mitochondrial genome for the banded linsang (appr. 10 million reads; Table 1; S1 Table of S1 Data; also in Taron et al. [19]).

#### Ancestral mitogenome enrichment

Sequence capture was performed using a 244k feature SureSelect microarray (Agilent), following the procedure described by Paijmans et al. [20]. A reconstructed ancestral mitogenome of the carnivoran family Felidae [12] was used to design bait sequences using 2 bp tiling. Although a more closely related mitogenome sequence has recently become available (spotted linsang, *P*. *pardicolor* [10]), this sequence was not published at the time the current study was initiated, thus the ancestral sequence assay was utilised [12]. Libraries were pooled prior to enrichment according to their target content as well as their molarity (S2 Table of S1 Data), to avoid libraries with high mitogenome content overwhelming samples with low mitogenome content on the array. After enrichment, approximately 30 million 75 bp paired-end reads were generated using the Illumina NextSeq 500 sequencing platform (S1 Table of S1 Data), using custom sequencing primers as needed for single-stranded libraries [15,21].

### Bioinformatic procedures

Raw sequences were trimmed using SeqPrep for paired-end sequence data (available from https://github.com/jstjohn/SeqPrep) and cutadapt v1.10 [22] for single-ended sequence data, both with default parameters. Reads shorter than 30 bp after trimming were discarded.

#### Mitogenome assembly & mapping

MITObim (Hahn et al. 2013) was used to assemble the *P*. *linsang* mitogenome, using the mitogenome from *P*. *pardicolor* (GenBank Acc. Nr. NC_024569 [10]) as reference sequence and a maximum mismatch value of 15%. After alignment, duplicates were identified according to both the 5’ and 3’ end mapping coordinates using MarkDuplicatesByStartEnd.jar (https://github.com/dariober/Java-cafe/tree/master/MarkDupsByStartEnd). To improve the accuracy of the resulting mitogenome further, we re-mapped the sequence data to the mitobim reference using the Burrows-Wheeler Aligner (BWA) v0.7.8 [23], with default values for seed length (32 bp) and mismatch values (0.04). Samtools v1.19 [24] was used to remove reads with a mapping quality < Q30. Duplicates were again identified and removed with MarkDuplicatesByStartEnd.jar. The final consensus banded linsang mitogenome sequence was then called from the mapped data, using a minimum read depth of 3x and a 90% majority rule for base calling. By using this stringent base calling strategy, any positions with inadvertent enrichment of nuclear copies of mitochondrial origin (numts) should be detected and not incorporated into the final consensus. Annotation was performed manually in Geneious v7.0 [25], using the published mitogenome sequence of the spotted linsang *P*. *pardicolor* (GenBank Acc. Nr. NC_024569 [10]) as reference. For linsang samples not selected for de-novo assembly, shotgun and capture sequences were mapped to the new mitobim-assembled banded linsang mitogenome, using the same mapping tools and parameters as described above.

#### Sliding window analysis

Sliding window sequence identity between the ancestral bait sequence and the *P*. *linsang* sequence was determined using a custom perl script, in 60 bp windows with a step-size of 30 bp. Due to insertion/deletion differences between the ancestral mitogenome and the linsang in the rRNA’s and the control region, this analysis excluded those regions and was restricted to the region between 2–15 kb. Sequencing depth in each window was recovered using bedtools v2.23 [26]. To assess the impact of sequence divergence on capture efficiency, we compared the shotgun data from individual PLI-12 to the capture data from the same individual. For this we investigated the relative proportions of the number of 60 bp windows and sequencing depth (capture/shotgun) at different levels of sequence divergence from the ancestral bait sequence. These metrics were plotted using ggplot2 [27] using the statistical programming environment R (http://www.cran.r-project.org).

#### Sequence alignment

Linsang sequences were aligned using ClustalW [28] as implemented in Geneious v7.0, with default parameters. For interspecific analyses, a second alignment was generated for the fossil calibration analysis (see below) following the same procedure, including the two most divergent banded linsang sequences (one from each matriline; PLI-5 and PLI-20) and 21 additional Feliformia species (S1 Data). All alignments were checked for internal stop codons using the vertebrate mitochondrial translation table in Geneious, to further ensure no numt sequences were integrated in the mitochondrial genome sequences. Furthermore, all columns with missing data were removed from the alignment.

#### Phylogenetic analysis

Four phylogenetic analyses were conducted, which are briefly described below (see S1 Text of S1 Data and S3 and S4 Tables of S1 Data for additional details):

- *Analysis 1* involved maximum-likelihood analysis using RaXML v8.2.4 [29] of representative mitochondrial sequences from the Feliformia clade, with the aim of reconstructing their phylogeny and determining the phylogenetic position of the linsang sequences.
- *Analysis 2* involved analysis of the Feliformia dataset using BEAST v1.8.2 [30], with the aim of estimating the divergence time of banded and spotted linsangs, and the basal divergence of the sampled haplotype lineages within the banded linsang, using a total of eight fossil calibrations (S4 Table of S1 Data). The latter was achieved by the inclusion of two sequences representing the maximal phylogenetic divergence observed within the sampled specimens based on prior exploratory analyses.
- *Analysis 3* involved analysis of all eight banded linsang sequences in BEAST, with the aim of estimating coalescence times of the sampled mitochondrial lineages, based on the tMRCA estimated in Analysis 2.
- *Analysis 4* involved generating a median-joining network for the banded linsang samples using PopArt v1.7 [31].

## Results

### High quality mitogenome assembly for the banded linsang

Approximately 8 million reads of shotgun data for sample PLI-12 were generated in order to assemble the mitogenome of the banded linsang with MITObim [32]. We recovered 55,442 unique reads using mitobim after 24 iterations. Conventional mapping (BWA) of the shotgun data to the mitobim sequence resulted in 53,797 unique reads (Table 1; S1 Table of S1 Data), which were then used to generate the consensus mitogenome sequence. Using this approach, 99% of the mitogenome was reconstructed, with an average sequencing depth of 156x. We found that the banded linsang mitogenome had 88% sequence similarity to the published mitogenome sequence of the spotted linsang. The annotated banded linsang mitogenome sequence has been deposited in GenBank (accession number MT559410).

### Ancestral capture efficiency at variable sequence similarity

**Fig 2:**
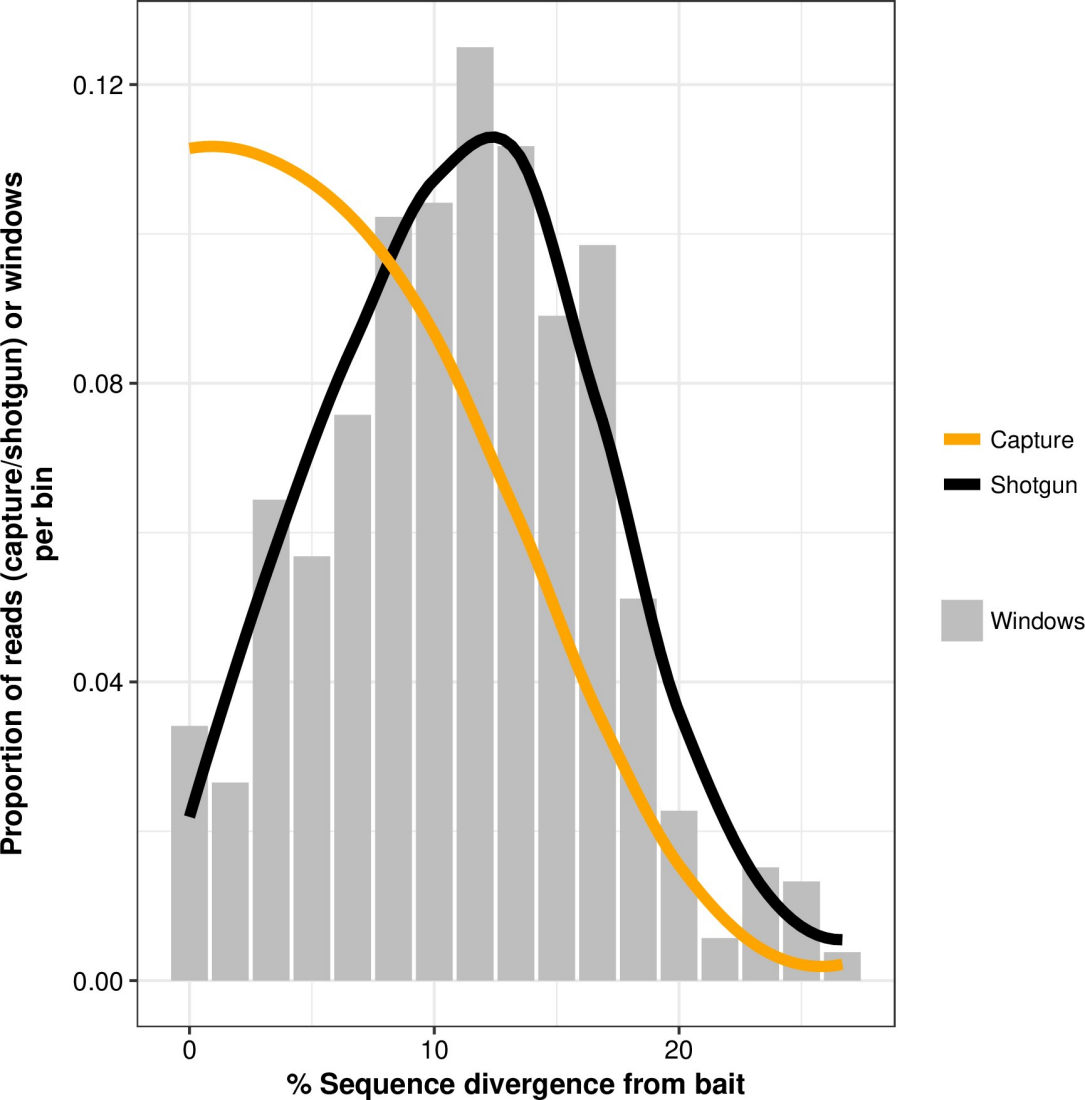
Impact of sequence divergence on capture. Impact of sequence divergence between bait and target on coverage in 60 bp sliding windows (step size of 30 bp). The proportion of the number of windows per sequence divergence bin is given by the grey bars; trendlines (loess smooth regression) for the proportion of unique mapped reads per sequence divergence bin are shown separately for captured libraries (orange) and the shotgun library (black). The shotgun library shows no bias towards/against windows with high or low divergence, whereas the capture libraries show a very clear bias towards high identity windows.

In addition to the complete mitogenome, we furthermore recovered partial mitochondrial genomes for an additional 7 banded linsangs (Table 1): between 50–90% of these mitogenome were retrieved with high sequencing depth (39-275x; Table 1). Regions with little to no sequence divergence from baits showed proportionally higher read depth when compared to the shotgun data (Fig 2; S1 Fig of S1 Data). The uneven coverage along the mitogenome following ancestral capture thus likely reflects local sequence divergence between bait and target, a general property of hybridization capture that has been observed in multiple studies [e.g. 12,33–35]. The de-novo assembled banded linsang mitogenome showed between 0% and 27% sequence divergence to the ancestral bait sequence, with a mean of 12%.

### Linsang phylogeny

**Fig 3:**
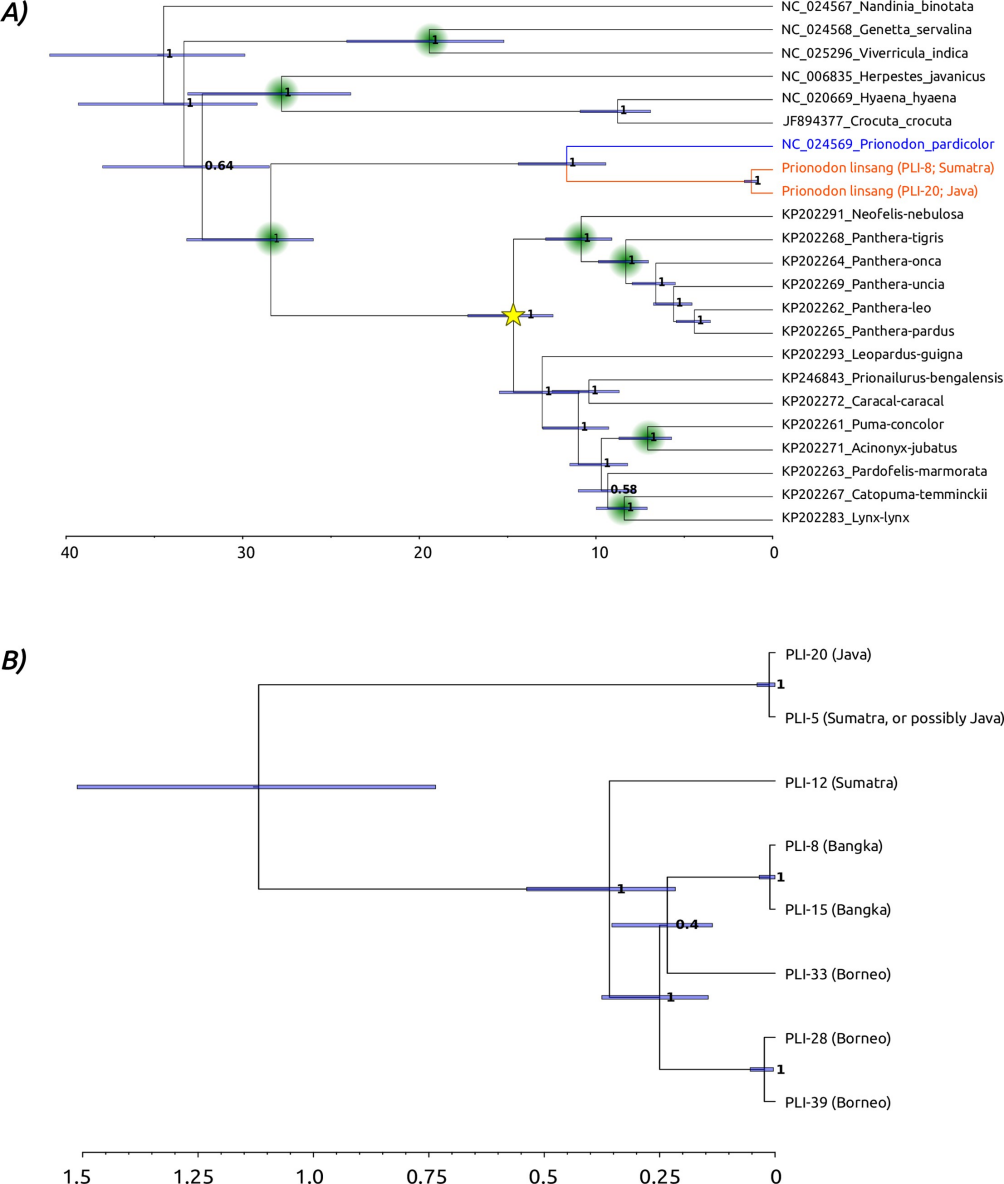
Linsang phylogenetic structure. A) Calibrated interspecific mitogenome phylogeny including a range of feliformia species. Calibrated nodes are indicated with green circles (S4 Table of S1 Data), and the ancestral bait sequence used for capture is indicated with a yellow star. Colours for the linsang taxa correspond to the distribution displayed in Fig 1. B) Calibrated mitogenome phylogeny of the banded linsang. On both panels, node labels indicate the Bayesian Posterior Probability, blue node bars indicate the 95% credibility interval of the posterior sampling of the node. Bottom axis indicates the age in millions of years.

Maximum likelihood analysis of mitochondrial sequences (23 sequences, 8001 bp) representing the Feliformia clade recovered the monophyly of the spotted linsang and the banded linsangs, and their position as sister to the Felidae clade, with 100% bootstrap support (S2 Fig of S1 Data). The median estimated age for the divergence of the two linsang species was 11.76 million years (Ma; 95% credibility interval [CI]: 9.44–14.40 Ma; Fig 3A). The median estimated age for the basal divergence of the banded linsang lineages was 1.22 Ma (95% CI: 0.85–1.60 Ma; Fig 3A). The intraspecific phylogeny (8 sequences, 8107 bp; Fig 3B; S2 Text of S1 Data) and MJ network (S3 Fig of S1 Data) reveal two divergent matrilines among the sampled banded linsang. Based on the estimated basal divergence of the banded linsang haplotype lineages, all other coalescence events among the eight sampled haplotypes are estimated to have occurred within the last ~500,000 years (Fig 3B).

## Discussion

Using a combination of shotgun sequencing and ancestral sequence capture, we were able to recover a complete mitogenome and seven additional partial mitogenomes of the banded linsang from historical museum specimens. This represents the first intraspecific mitochondrial dataset for this species, and provides novel insights into their evolution.

Phylogenetic analysis supports a deep divergence within the Asiatic linsangs, with the two recognised species coalescing around 12 Ma. This deep divergence between the spotted and banded linsang has been shown previously using short mtDNA sequences [36,37]. It is notable that this divergence approximates that found among all living felid species [38]. Unfortunately, our small sample size and the questionable provenance of one of the Sumatran samples precludes any robust interpretation of the intraspecific population structure of the banded linsang. The potential presence of two divergent matrilines within the banded linsangs could serve as a basis for further, more thorough, investigation of the population structure using more samples and more genetic markers.

The problematic provenance of one of the Sumatran samples highlights both the advantages but also the difficulties of working with museum samples. As methodologies to mediate the less-than-favourable sample quality develop further and further, museum collections become an increasingly accessible and important sample source, providing access to genetic material of rare or extinct lineages that are otherwise not readily available. However, the provenance of museum samples is sometimes ambiguous, specifically regarding uncertainties about the geographic origin of the sample. Despite these issues, museum samples will remain a vital resource for rare species such as the Asiatic linsang.

Our study also provides further useful information on the practical application of ancestral capture. As the Felidae clade for which we reconstructed the ancestral mitogenome sequence is sister to the linsang lineage, we show that ancestral capture can be applied to enrich not only a range of descendant [12] as well as sister taxa (this study). Pooling of multiple samples in the same capture experiments will greatly leverage the sample throughput and cost-efficiency. However, in previous studies, pooling multiple libraries in the same capture experiment has sometimes led to uneven representation of each library in the post-capture pool [e.g. 39]. We find that pooling samples on the array according to their target content (based on low level shotgun sequencing) as well as their molarity, provided a relatively equal representation of reads per library in the post-capture pool (S1 Table of S1 Data). We therefore recommend this approach for pooling samples of variable sample quality in the same capture experiment in order to increase throughput and to decrease per-sample costs. While we carried out the capture on Agilent SureSelect arrays, this pooling strategy is likely to be beneficial to in-solution capture approaches as well.

Previous studies have found a strong correlation between enrichment efficiency and bait-to-target similarity [e.g. 12,33,34,40], while successful enrichment has been reported for up to 40% sequence divergence between bait and target [e.g. 41]. The maximum sequence divergence between our bait and target sequences was less than 30%. The large difference we observed between highly successful recovery at high-similarity regions (more than 1000x read depth in regions <10% sequence divergence) but low recovery at low-similarity regions (down to 0x depth in regions >20% sequence divergence) suggests a strong bias towards the former, in our case severe enough to lose the latter regions entirely. Some of this bias is expected due to the increased mismatches between bait and target; however, the lack of recovered data at higher levels of sequence divergence is not expected. It is possible that this may have resulted from a template abundance bias in post-capture amplification steps, and/or the two consecutive capture rounds. Although a single capture round has been shown to be less effective for overall enrichment rates compared with two consecutive capture rounds for degraded samples [42–44], the bias in recovery of low-similarity regions we observed may be partially reduced when only a single round of capture is used. An alternative solution could potentially be to split the capture baits into high- and low-identity pools, and capture the library separately with each bait pool—although not every bait preparation method will lend itself for this strategy (e.g. when preparing home-made baits from PCR products), and this also requires prior knowledge of local sequence divergence which may not be available.

Despite the inability of our ancestral capture strategy to recover more divergent mitochondrial regions in this experiment, overall we were able to recover a sufficient portion of the mitochondrial genome for phylogenetic and phylogeographic analyses. Thus, even without further optimisation, this approach is tenable for many applications including phylogenetic, phylogeographic and conservation genetic investigations of rare taxa. Furthermore, it is notable that this data can be generated rapidly and at relatively low cost, including for historical museum specimens, and is applicable even to the sister taxon of the ancestral bait sequence in addition to the descendent clade. Ancestral capture is thus a useful tool for genetic investigation of species which lack sequence information from close relatives.

## Supporting information

S1 Data (PDF)
