# Supplementary material for: Ancestral mitogenome capture of the Southeast Asian banded linsang
Source: PLoS One. 2020 Jun 30;15(6):e0234385. doi: 10.1371/journal.pone.0234385 (PMC7326216; doi:10.1371/journal.pone.0234385)
Supplement: S1 Data — (PDF) [file pone.0234385.s001.pdf]

Ancestral mitogenome capture of the Southeast Asian banded linsang

Johanna L.A. Paijmans[1,2,§], Axel Barlow[1*,†], Kirstin Henneberger[1], Joerns Fickel[1,2], Michael Hofreiter[1], Daniel W.G. Foerster[2,*]*

[1] Institute for Biochemistry and Biology, University of Potsdam, Karl-Liebknecht-Str. 24-25, 14476 Potsdam, Germany
[2] Leibniz Institute for Zoo- and Wildlife Research, Alfred-Kowalke-Str. 17, 10315 Berlin, Germany

* Corresponding authors: JLAP: paijmans.jla@gmail.com, AB: axel.barlow.ab@gmail.com, DWF: dwgfoerster@gmail.com

[§] Present Address: Department of Genetics & Genome Biology, University of Leicester, Leicester LE1 7RH, UK
[†] Present Address: School of Science and Technology, Nottingham Trent University, Clifton Lane, Nottingham NG11 8NS, UK

**Supporting Figure S1:** Sequence coverage & capture efficiency per sample	2
**Supporting Figure S2:** RaXML tree of Feliformia	3
**Supporting Figure S3:** Median-joining network of banded linsangs	3
**Supporting Table S1:** Summarised sequence statistics	4
**Supporting Table S2:** Pooling strategy for hybridisation capture.	5
**Supporting Table S3:** Details of substitution models used	6
**Supporting Table S4:** Calibration priors used for BEAST analysis	7
**Supporting Text S1:** Supporting methods for phylogenetic analysis	8
**Supporting Text S2:** Banded linsang alignment utilised in phylogenetic analysis	9
**Supporting References**	15

Supporting Figure S1: Sequence coverage & capture efficiency per sample. *Coverage along mitogenome for each library in 60 bp sliding, with colours indicating the pairwise similarity between bait and target (Red = high similarity, yellow is low similarity). The bottom sample is the high coverage mitogenome generated using shotgun sequencing.*

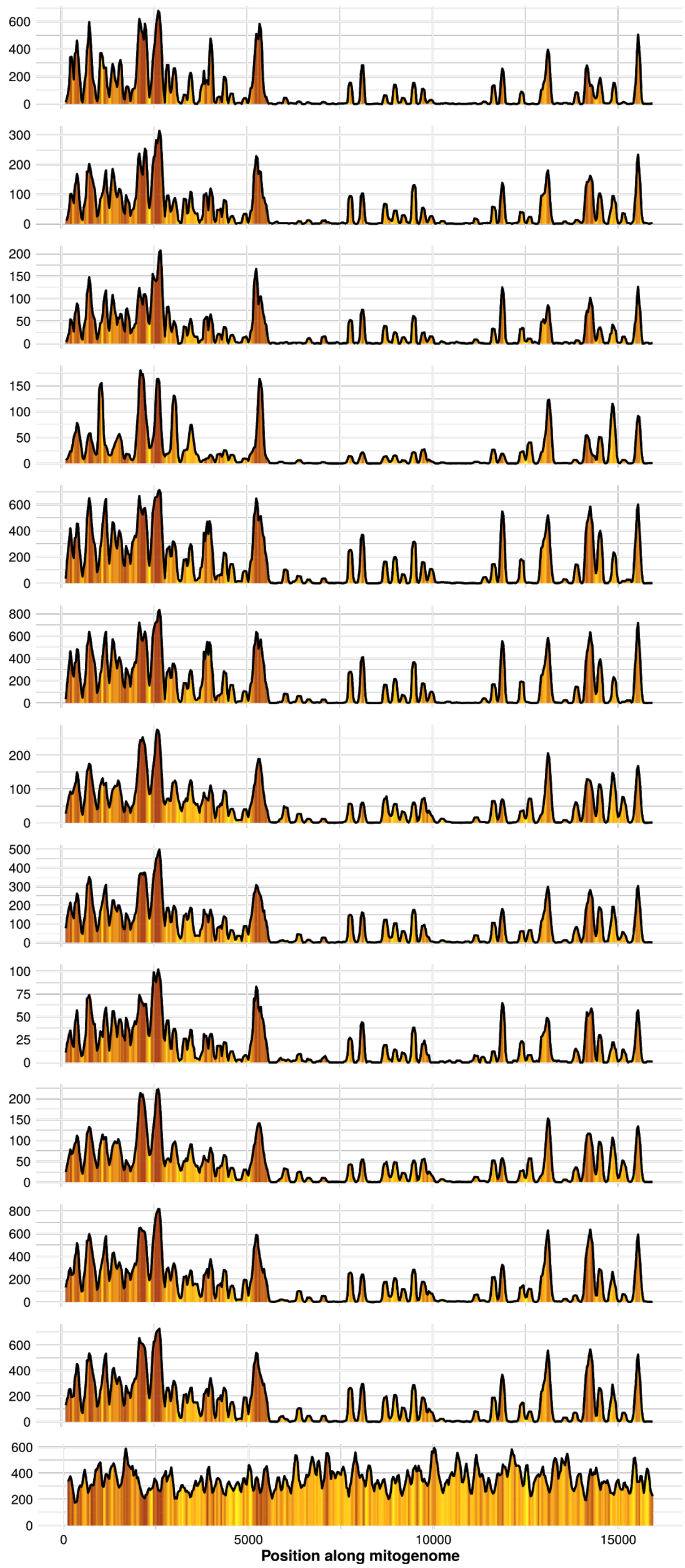

Supporting Figure S2: RaXML tree of Feliformia. *RaXML maximum-likelihood tree of Feliformia, including the spotted linsang and two most divergent banded linsang lineages (PLI-8 & PLI-20). Colours for the linsang taxa correspond to the distribution displayed in Figure 1. Node label indicate the bootstrap support for that clade.*

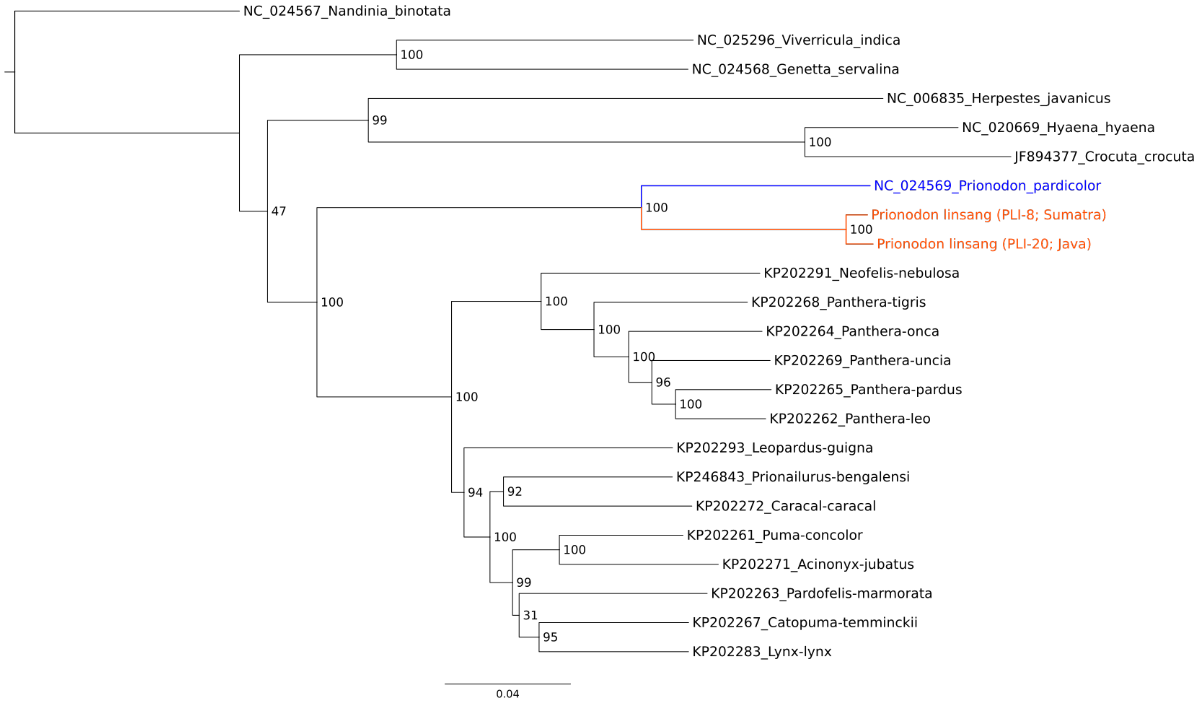

Supporting Figure S3: Median-joining network of banded linsangs. *Number of substitutions for each edge are indicated between brackets. Edge lengths reflect the number of substitutions, except for the long branch between PLI-20/PLI-5 and the remaining samples, which is compressed to improve figure clarity.*

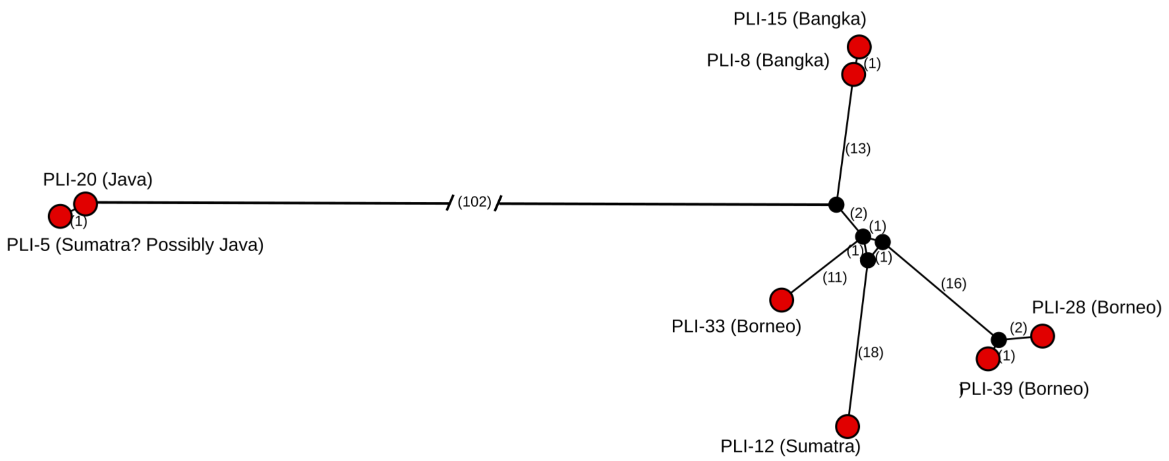

Supporting Table S1: Summarised sequence statistics*. Shotgun data was generated to establish endogenous content for most samples (except for 5 libraries), by mapping shotgun data to the spotted linsang (*P. pardicolor*). Mappable reads indicates the number of reads after trimming and merging of the raw sequence reads. For some individuals, two libraries were prepared. "_DS" indicates a double-stranded library was prepared (Henneberger et al., 2019), all other libraries were prepared using single-stranded library preparation (Gansauge & Meyer, 2013).*

High-coverage data for de-novo assembly PLI-12

name	MAPPABLE	READS (mapped to *P. linsang*)	UNIQUE READS (mapped to *P. linsang*)	basepair	depth	mitogenome covered ≥3x
PLI-12_L1	6,178,108	60,096	53,797	2,633,567	157.09	99.12%

	Shotgun data			Capture data					Merged libraries per individual		
Sample ID	MAPPABLE	UNIQUE READS (mapped to *P. pardicolor*)	%_on-target	MAPPABLE	UNIQUE READS (mapped to *P. linsang*)	duplication	mapped_BP	depth	MAPPABLE	UNIQUE READS (mapped to *P. linsang*)	mitogenome covered ≥3x
eBLK-5-10-15-C_L1	89,007	1	0.001%	52,321	62	94.61%	3,512	0.21			
eBLK-5-10-15-C_L2	161,460	0	0.000%	33,978	0	0.00%	0	0			
eBLK-5-10-15-D_L1	150,223	0	0.000%	29,977	1	91.67%	34	0			
eBLK-5-10-15-D_L2	204,452	0	0.000%	105,113	106	94.84%	5,598	0.33			
liBLK-11-11-15-B_L1	179,783	0	0.000%	105,113	2	95.83%	150	0.01			
liBLK-11-11-15-B_L2	174,386	0	0.000%	37,478	1	0.00%	51	0			
liBLK-2-11-15_L2	139,339	0	0.000%	34,221	0	0.00%	0	0			
liBLK-20-10-15_L1	70,382	0	0.000%	309,427	512	93.92%	26,158	1.56			
eB-5-10-2015-B_DS	42,742	0	0.000%	3,597	0	0.00%		0			
LiBLK-7-10-2015_DS	2,143	0	0.000%	166	0	0.00%		0			
eBLK-5-10-15-D	-	-	-	105113	106	94.84%	5598	0.33			
PLI-12_L1	337,269	506	0.150%	189,239	3,727	97.57%	208,292	12.42			
PLI-12_L2	292,628	473	0.150%	299,949	6,296	97.67%	368,539	21.98			
PLI-20_L1	616,948	61	0.010%	1,656,101	18,395	98.08%	1,211,436	72.26	3,695,331	34,277	75.30%
PLI-20_L2	699,002	65	0.009%	2,039,230	19,767	98.44%	1,299,678	77.53			
PLI-33_L1	397,078	38	0.010%	230,720	2,156	97.64%	140,057	8.35	1,955,173	12,231	69.27%
PLI-33_L2	441,104	50	0.011%	752,399	10,162	97.68%	739,295	44.1			
PLI-8_L1	653,197	164	0.025%	1,202,774	17,887	97.96%	1,317,430	78.59	2,192,420	33,015	85.76%
PLI-8_L2	579,309	141	0.024%	989,646	17,235	97.73%	1,264,500	75.43			
PLI15_DS	531,662	47	0.009%	2,435,381	23,842	97.73%	2,014,252	120.15	2,435,381	23,842	68.88%
PLI19_DS	240,503	2	0.001%	2,582,596	1,614	97.92%	80,792	4.82	2,582,596	1,614	NA
PRP1_DS	863,432	50	0.006%	1,416,010	141	98.31%	8,968	0.53	1,416,010	141	NA
PLI-45	-	-	-	26,304	1	0.00%	38	0	26,304	1	NA
PLI-39	-	-	-	2,456,739	42,510	97.67%	3,635,492	216.86	2,456,739	42,510	91.63%
PLI-28	-	-	-	2,954,993	55,243	97.52%	4,513,790	269.25	2,954,993	55,243	90.02%
PLI-5	-	-	-	1,820,963	13,984	98.73%	645,010	38.48	1,820,963	13,984	50.95%

Supporting Table S2: Pooling strategy for hybridisation capture. *Samples were pooled taking both their concentration as well as their target content into account (see Supporting Table S1), in order to prevent high target-content samples to outcompete low target-content samples on the array. Samples indicated with an asterisk (*) were not shotgun sequenced prior to capture, so the endogenous content was estimated.*

Sample	Concentration (ng/µl)	Peak	Estimated nM	Estimated target content (% mapped to *P. pardicolor)*	Ratio of sample in total target content on the array	Reversed Ratio of run	Final concentration in pool (nM)	Total volume (µl)	Volume per library pooled (µl)	Estimated amount of DNA (ng)	Estimated amount of target DNA (ng)	Estimated amount of target DNA per individual (ng)
eBLK_5.10.15_B_DS	10.7	128	126.7	0.000%	-	-	1.000	168	1.326	14.19	-	-
eBLK_5.10.15_C_Lib1	6.22	156	60.4	0.001%	-	-	1.000	168	2.781	17.30	-	-
eBLK_5.10.15_C_Lib2	6.04	168	54.5	0.000%	-	-	1.000	168	3.084	18.63	-	-
eBLK_5.10.15_D_Lib1	11.1	168	100.1	0.000%	-	-	1.000	168	1.678	18.63	-	-
eBLK_5.10.15_D_Lib2	10.3	168	92.9	0.000%	-	-	1.000	168	1.809	18.63	-	-
liBLK_11.11.15_B_Lib1	11.1	167	100.7	0.000%	-	-	1.000	168	1.668	18.52	-	-
liBLK_11.11.15_B_Lib2	9.5	165	87.2	0.000%	-	-	1.000	168	1.926	18.30	-	-
liBLK_2.11.15_Lib2	6.88	167	62.4	0.000%	-	-	1.000	168	2.691	18.52	-	-
liBLK_20.10.15_Lib1	8.96	162	83.8	0.000%	-	-	1.000	168	2.005	17.96	-	-
LiBLK_7.10.15_DS	10.9	123	134.3	0.000%	-	-	1.000	168	1.251	13.64	-	-
eBLK_5.10.15_D	9.74	158	93.4	0.000%*	-	-	1.000	168	1.799	17.52	-	-
liBLK_20.01.2016	19.7	156	191.3	0.000%*	-	-	1.000	168	0.878	17.30	-	-
PLI-12_Lib1	6.32	181	52.9	0.150%	0.328	3.051	0.254	168	0.807	5.10	0.0077	0.0164
PLI-12_Lib2	4.9	173	52.9	0.162%	0.353	2.832	0.283	168	1.109	5.43	0.0088	
PLI-20_Lib1	25.2	194	196.8	0.010%	0.022	46.301	4.630	168	3.952	99.60	0.0098	0.0195
PLI-20_Lib2	19.4	191	153.9	0.009%	0.020	49.230	4.923	168	5.374	104.26	0.0097	
PLI-33_Lib1	4.76	184	39.2	0.010%	0.021	47.837	4.784	168	20.503	97.60	0.0093	0.0192
PLI-33_Lib2	6.02	195	46.8	0.011%	0.025	40.387	4.039	168	14.505	87.32	0.0099	
PLI-8_Lib1	12.6	197	96.9	0.025%	0.055	18.233	1.823	168	3.161	39.83	0.0100	0.0197
PLI-8_Lib2	10.8	191	85.7	0.024%	0.053	18.809	1.881	168	3.688	39.83	0.0097	
PRP-1_DS	49	193	384.7	0.006%	0.013	79.054	15.811	168	6.905	338.35	0.0196	0.0196
PLI-15_DS	48.8	205	360.7	0.009%	0.019	51.785	10.357	168	4.824	235.42	0.0208	0.0208
PLI-19_DS	122	186	993.8	0.001%	0.002	550.501	110.100	168	18.612	2270.67	0.0189	0.0189
PLI-28	9.3	214	65.8	0.010%*	0.022	45.779	9.156	168	23.360	217.25	0.0217	0.0217
PLI-39	10.3	240	65.0	0.010%*	0.022	45.779	9.156	168	23.655	243.65	0.0244	0.0244
PLI-45	52	141	558.8	0.010%*	0.022	45.779	9.156	168	2.753	143.14	0.0143	0.0143
PLI-5	16.8	168	151.5	0.010%*	0.022	45.779	9.156	168	10.152	170.55	0.0171	0.0171

Supporting Table S3. Details of substitution models used. *Results of PartitionFinder analysis and details of substitution models used. Base frequencies were estimated in all cases.*

**Analysis**	**Partition**	**Composition**	**BIC model**	**Utilised model**
Analysis 1	p1	tRNA-Tyr, ND4L, tRNA-Cys, tRNA-Phe, tRNA-Leu, tRNA-Met, tRNA-Asn, tRNA-Val, tRNA-Trp, tRNA-Ser1, tRNA-Ala, tRNA-Leu1, tRNA-Thr, tRNA-Ile, l-rRNA, s-rRNA, tRNA-Pro, tRNA-Ser, tRNA-Gln, tRNA-Glu	N/A	GTR+CAT/G
	p2	CYTB, ND1, ATP8, ND3, COX2, ND5	N/A	GTR+CAT/G
	p3	CR, ND6, ND4, ND2	N/A	GTR+CAT/G
	p4	COX1, ATP6, COX3	N/A	GTR+CAT/G
Analysis 2	p1	ND4L, l-rRNA, s-rRNA, tRNA-Ala, tRNA-Asn, tRNA-Cys, tRNA-Gln, tRNA-Glu, tRNA-Ile, tRNA-Leu, tRNA-Leu1, tRNA-Met, tRNA-Phe, tRNA-Pro, tRNA-Ser, tRNA-Thr, tRNA-Trp, tRNA-Tyr, tRNA-Val	GTR+I+G	GTR+G
	p2	ATP6, ATP8, COX1, COX2, COX3, CYTB, ND1, ND3, ND5, tRNA-Ser1	GTR+I+G	HKY+I+G
	p3	CR, ND2, ND4, ND6	HKY+I+G	HKY+G
Analysis 3	p1	ATP6-CDS, COX1-CDS, COX2-CDS, COX3-CDS, ND3-CDS, TRNA-Leu1, TRNA-Ser1, l-rRNA, rep_origin, s-rRNA, tRNA-Ala, tRNA-Glu, tRNA-Ile-GLN, tRNA-Leu, tRNA-Met, tRNA-Phe, tRNA-Ser, tRNA-Thr, tRNA-Trp, tRNA-Tyr, tRNA-Val	TrN	TrN
	p2	ATP8-CDS, CYTB-CDS, D-loop, ND1-CDS, ND2-CDS, ND4-4L-CDS, ND5-CDS, ND6-CDS, tRNA-Asn, tRNA-Cys, tRNA-Pro	TrN	TrN

Supporting Table S4: Calibration priors used for BEAST analysis. *Fossil calibrations and implemented priors for the interspecific BEAST analysis.*

**Fossil**	**Fossil constraint**	**Calibration prior**	**Citation**
Genetta fossil: 11.2M	Minimum 11.2M	Uniform: 50M – 11.2M	[1,2]
Hyaenid & Herpestid fossil: 16.4M	Minimum 16.4M	Uniform: 50M – 16.4M	[1,2]
Felidae stem fossils, *Prionodon* fossils	Minimum 26M	Uniform: 50M – 28M	[1,2]
Lynx fossil: 5.3M	Minimum 5.3M	Uniform: 10M – 5.3M	[1,2]
Acinonyx fossils: 3.8M	Minimum 3.8M	Uniform: 10M – 3.8M	[3,4]
Caracal & Serval fossils: 3.8M	Minimum 3.8M	Uniform: 16M – 3.8M	[4]
Oldest *Panthera* fossil: 3.8M	Minimum 3.8M	Uniform: 16M – 3.8M	[4,5]
Oldest *Panthera tigris* fossil: 1.5M	Minimum 3.5M	Uniform: 10M – 1.5M	[5]

Supporting Text S1: Methods for phylogenetic analysis

Alignment columns containing missing data were excluded from each dataset prior to phylogenetic analysis. For each analysis, optimal partitions and, where appropriate, substitution models were selected from all possible combinations of genes, tRNAs and the control region under the Bayesian Information Criterion using PartitionFinder v1.1.1 [6]. For Analysis 1, only the GTR+G model was considered, and for Analyses 2 and 3 all substitution models available in BEAUti v.1.8.2 (part of the BEAST v1.8.2 distribution [7]) were considered.

For Analysis 1, we used RaxML-HPC BlackBox v8.2.10 [8] on the CIPRES gateway [9]to carry out 250 maximum likelihood bootstrap replicates using the computationally fast GTR+CAT model for each partition, which approximates the GTR+G model. The maximum likelihood tree was then estimated using the GTR+G model for each partition. The African palm civet (*Nandinia binotata*) served as outgroup to root the tree.

Analysis 2 was carried out using BEAST v.1.8.2, after generating the input .xml file in BEAUti v1.8.2. A birth-death speciation model was used for the tree prior. Optimal substitution models indicated by PartitionFinder failed to achieve convergence, and so the models were simplified by reducing the number of free parameters (Supporting Table S3). Nucleotide frequencies were estimated in all cases. Separate uncorrelated lognormal relaxed clock models were specified for each partition, and their respective substitution rates estimated within uninformative uniform priors from 0% to 20% per million years. Time calibration was based on a scheme presented by Paijmans et al. [10] (see Supporting Table S4) involving uniform priors on the ages of nine nodes with minimum ages representing the age of the oldest reliable fossil representative of each clade and maximum ages based on current palaeontological and fossil-calibrated phylogenetic knowledge. This approach provides hard minimum ages that are compatible with the fossil record, but assigns equal probability to considerable older divergence times, thus accounting for potentially incomplete fossil records. The monophyly of each calibration clade was enforced, based on the results of Analysis 1, as well as the position of the African palm civet as sister to the Feliformia clade. The MCMC chain ran for sufficient length to achieve convergence and adequate sampling (ESS > 200) of all parameters, determined using the program Tracer as distributed with BEAST v1.8.2. The maximum clade credibility tree was extracted from the posterior sample, nodes heights scaled to the median of the posterior sample, and annotated with various summary statistics using the program TreeAnnotator, and visualised in FigTree v1.4.2.

For analysis 3, divergence times among the eight sampled *P. linsang* haplotypes were estimated by placing a normal prior to the tree root with a mean age of 1.2175 Ma and standard deviation of 0.1927 Ma, based on the results of Analysis 2. Upper and lower hard limits were enforced on the root age of 2.5 Ma and 0.5 Ma, respectively. A Bayesian skyline plot model was used as the tree prior. Strict clock models were used for each partition, following preliminary runs using lognormal relaxed clock models that failed to reject zero substitution rate variation among the sampled lineages. All other aspects of the analysis were as described for Analysis 2.

Supporting Text S2: Banded linsang alignment utilised in phylogenetic analysis

```
>PLI-12_mitobim-ref__3x-90p_noNs
TCCAAGTTAATGTAGCTTAAACCATAAAGCAAGGCACTGAAAATGCTTAGATGAGTTGTAATAACTCCATAAACATAAAGGTTTGGTCCTGGCCTTTCTATTAGTTATTAGTAGGATTACACATG
CAAGCCTCCGCATCCCGGTGAAAATACCCTCTATGTCATTCATGACCCAAAGGAGTAGGCATCAAGCACACAACCACTGTAGCTCATGACGTCTTGCAAAGCCACACCCCCACGGGATACAGCAG
TGATAAAAATTAAGCTATGAATGAAAGTTCGACTAAGCCATACTAAACTTAAGGGTTGGTAAATTTCGTGCCAGCCACCGCGGTCATACGATTAACCCGAACTAATAGATCTACGGCGTAAAGCG
TGTTAAAGAAATAAGCCTTACTAGAGTTAAGTCTTAACTAACAGAAAGTAGTCTAATATCTCTAACTACACGATAGCTAAGATCCAAACTGGGATTAGATACCCCACTATGCTTAGCCCTAAACC
TAGATGACTTTACCCAACAAAGTTATCCGCCAGAGAACTACTAGCAACAGCTTAAAACTCAAAGGACTTGGCGGTGCTTTATATCCCTCTAGAGGAGCCTGTTCTATAATCGATAAACCCCGATA
TACCTCACCACTCCTTGCTAATCCAGTTTATATACCGCCATCTTCAGCAAACCCTTAAAAGGAATAAAAGTAAGCACAAGTATTTTTAACACAAAAAAGTTAGGTCAAGGTGTAACCTATGGAGT
GGGAAGTAATGGGCTACATTTTCTGATTATGAAATTAAGAACTAAAGGAGGATTTAGCAGTAAATTGAGAATAGAGTGCTCAATTGAATCGGGCCATGAAGCACGCACACACCGCCCGTCACCCT
CCTCAAGTGACAACCTATAAATAACATAATTATCCCATAAAATCACAAGAGGAGATAAGTCGTAACAAGGTAAGCATACTGGAAAGTGTGCTTGGATAACCAAGATGTAGCTTAAGTAAAGCATC
TGGCCTACACCCAGAAGATTTCATATTAAACTGAACATCCTGAGCTAAAACTAGCCCAAACAACTACAAACTCAACTTTCACTCAACAATAAATCAACCATTTAATTACTAAAATTAAAAGTATA
GGAGATAGAAATTTAACTTGGCGCCATAGAGATAGTACCGCAAGGGAAAGATGAAAGAAGTATTTAAAGCACCACACAGCAAAGATTATCCCTTGTACCTTTTGCATAATGAATCAGCTAGAACG
CCCTAGCAAAGAGAACTTAAGCTAGACTCCCCGAAACCAGACGAGCTACCTGTGAACAATCTACATGGGATGAACTCATCTATGTCGCAAAATAGTGAGAAGATTTACAGGTAGAGGTGAAAAGC
CTAACGAGCCTGGTGATAGCTGGTTGCCCAGGATAGAATTTTAGTTCAACTTTAAACTTACCCGCAAATCTCCCTTAAAATTCTAATGTAAGTTTAAAATATAATCTAAAAAGGTACAGCTTTTT
AGAACTAGATACAGCCTTTTTTAGTGAGTAAGCATAATTATTAACCATAGTCGGCCTAAAAGCAGCCATCAATTAAGAAAGCGTTCAAGCTCAACAGTTAAAATATCTTAATATCAAAAGTAATT
ATACAATAGAAGCAATAATGCTGATATGAGTAACAAGAAATACTTCTCCTTGCATAAGCTTATATCAGAGCGGATAACCACTGATAGTTAACATCAAGATATGAAAAACCTACCTATAAATTCAA
TATCAAGTTAATTGTTACCCAACACAGGCATGCAATCAAGGAAAGATTAAAAGAAGTAAAAGGAACTCGGCAAACACAAGCCCCGCCTGTTTACCAAAAACATCACCTCTAGCATTTCTAGTATT
AGAGGCACTGCCTGCCCAGTGACATTAGTTAAACGGCCGCGGTATCCTGACCGTGCAAAGGTAGCATAATCACTTGTTCCCTAAATAGGGACTCGTATGAATGGCCACACGAGGGCTTTACTGTC
TCTTACTTCCAATCCGTGAAATTGACCTTCCCGTGAAGAGGCGGGAATGTGACAATAAGACGAGAAGACCCTATGGAGCTTTAATTAACTAACTCAGAGAGATTACTTTAACATACGACAACAAA
CCTCTATATGAGTTAGCAATTGGGTTGGGGTGACCTCGGAGAACAAAAAAACCTCCGAGTGATTTTAATCTAGACTAACCAGTCAAAAGTATTACATCACTTATTGATCCAAAAACTTGATCAAC
GGAACAAGTTACCCTAGGGATAACAGCGCAATCCTATTTTAGAGTCCATATCGACAATAGGGTTTACGACCTCGATGTTGGATCAGGACATCCCGATGGTGCAGCAGCTATCAAAGGTTCGTTTG
TTCAACGATTAAAGTCCTACGTGATCTGAGTTCAGACCGGAGTAATCCAGGTCGGTTTCTATCTATTAAACAATTTCTCCCAGTACGAAAGGACAAGAGAAATAGGGCCTACCTTACCAAGACGC
CCTCAACTGTAGATGTACGGGTTAGTTAGGGTGGCAGAGCCCGGTAATTGCATAAAACTTAAACTTTTACAATCAGAGGTTCAATTCCTCTCCCTAACAATATGTTCATAATTAATATTCTATCA
CTAATCGTTCCAATCCTTCTCGCCGTAGCCTTCCTAACACTAGTTGAACGTAAAGTCTTAGGTTACATACAACTCCGCAAGGGACCAAACATTGTAGGACCATACGGTCTGCTCCAACCCATCGC
AGACGCCATTAAATTATTTACCAAAGAACCCCTGCGACCCCTAACATCCTCCATATCAATATTCATCATAGCACCAATCCTAGCCCTTCACTGCTCTAACCATATGAATCATAAATTTCATACTA
GCCATATAAGTTTAGCCGTATATTCCATTTTATGCCTCAAACTCCAAATATGCCTTAATTGGAGCTCTACGAGCCGTAGCCCAAACAATCTCATATGAAGTCACACTAGCTATCATTCTCCTATC
CGTGCTCTAATTACCACCCAAGAATATATATGGCTAATCTTTCCCGCATGACCCCTAGCCATAATATGATTTATCTCAACTCTAGCAGAAACCAACCGAGCCCCATTCGACCTTACAGAAGGAGA
GTCAGAACTCGTCTCTGGCTTCAATTAGAATTTATTCCTAACTATCTCCTTCCGAATTCGAGCATCATATCCCCGATTCCGATATGATCAACTTATACACCTATTATGAAAAAACTTCCTACCCC
TAACTCTAGCCCTATGTATATGACACGTATCCCTTCCCATCATAACTGCAAGTATTCCACCCCAAACATAAGAAATATGTCTGACAAAAGAGTTACTTTGATAGAGTAAATAATAGAGGTTTAAG
CCCTCTTATTTCTAGAGCTATAGGAATCGAACCCAATCCTAAGAATTCAAAAATCTTTGTGCTACCATATTACACCACGCTCTATAGTAAGGTCAGCTAAGTAAGCTATCGGGCCCATACCCCGA
AAATGTTGGTTTATACCCTTCCCGTACTAATTAAACCGCCTATTCTCATCATCATTATATCAACTGTAATCTCAGGGAAATTGTAAAGCTCTAACAATCTGAATCGGCTTTGAAATAAACATTCT
TATAAAAAAATTTAACCCCCGAGCTATAGAAGCAGCTACAAAATATTTCCTCACACAAGCAACCGCATCCATACTATTAATAATAGGAATTATCATCAAATAAACCCTCACCCCATAGCATCAAT
TATAATAACCACAGCCCTAGCAATAAAACTAGGTCTAGCCCCATTCCACTTCTGAGTACCCGAAGTAGCACAAGGAATTTCCCTATCCTCAAGTCTCATTCTATTAACATGACAAAAATACTTCT
AACAATAGCTATTACATCAGTTATCATCGGGGGCTGAGGAGGACTAAATCAAACTCAACTACGAAAAATCATAGCATATTCCTCAATCGCACACATATTCATACATAGCTCTTCTACCACAACAT
ATCCCTATCCCAAACATAAACAAAGCACCCTTGATCACCTCACTACTATTATCCCCACATACTAGCCATCACAGCACTACTCAATCTATACTTCTACATACGACTAACGTACGCCACAGCACTTA
CTATATTTCCCTCAGCTAAAACATAAAAATAACACTTTCAACTATACTCTCCCTCTGCCCCCATAATATCCATCCTGGATTAGAAGTTTAGGTTAGATAGACCAAGAGCCTTCAAAGCCCTAAGC
AAGCCTCACTGGCTTAACTTCTGAACTCCCATAAGGACTGCAAGAATCTATCTCACATCAATTGATTGCAAATCAAACACTTTAATTAAGCTAAGCCCTTACTAGATTGGTGGGATCCAACCCCA
CGAAACTTTAGTTAACAGCTAAACACCCTAATCAACTGGCTTCAATCTACTTCTCCCGCCGTCTAGAAAAAAAAGGCGGGAGAAGCCCCGGCAGCGTTAAAGCTGCTTCTTTGAATTTGCAATTC
AACATGAATATTCACCACAGGACTTGGTAAAAAGAGGATTAAACCTCTGTTCTTAGATTTACAGTCTAATGCTTTGCTCAGCCATTTTACCTATGTTCATAAACCGTTGATTATTTTCAACTAAC
CACAAAGATATCGGAACTCTATTCAATAGTAGAAGCCGGGGCAGGAACCGGATGAACAGTTCTAGAGGTAATTTAGCACATGCAGGAGCATCGTAGACACCACAATCATTAACATAAAACCTCCT
GCTATATCCCAATACCAAACACCTTTATTTGTCTGATCAGTCTTAATCACGCTGTTCTATTCGTGTGAGCTCACCATATGTTTACTGTAGGCATGGATGTGGACACACGAGCATATTTTACATCA
GCTACTATAATTATCGCAATTATGTAGTAGCCCATTTCCACTATGTATTATCAATAGGAGCAGTTTTCGCTATTATAGGAGGCTTTGTACATTGATTTCCTTTATTTGAAGAACTGCCTACGTAT
CTTTAAAGTAAGAAAGGAAGGAATCGAACCTCCTAGGACTGGTTTCAAGCCAATATCATAACCATTACACTCTGAGCGGTCCATCACTAGGACTATAAATCAAACAACTTTAATAGGCACACGTC
CAGGGTTATACTATGGTCAATGCTCAGAAATCTGTGGCTCAAACCATAGTTTTATACCTATCGTCCTTGAATTAGTACCATTAACATATTTCGAGAAACCCCTGAAATCCAGAACCTAAATCTGC
AATTACACTAAAACAACCTAATCCTTGAGAAAAAAAATGAACGAAAATCTATTCGCCTCTTTCACTACCCCAACGATAATAGGCTTGCCCATTGTTATCTTAATCGTATTATTATCCTAGTCCTA
CTAACAATCCTTGAATTCGCAGTAGCCCTAATTCAAGCCTATGTCTTTACCCTGCTAGTAAGCCTATACTTACATGATCATATATGTACCAATGATGACGAGATGTTATTCGAGAAAGCACGTTT
CAAGGCCACCACACACCCGTTGTTCAAAAAGGATTACGATATGGAATAATCCTATTTATTGATCTGAAGCTGAGCATTTTATCATTCAAGCTCCAGATTAGGAGGTTGCTGACCCACAGGCATTA
TTCCTCTAATCCTCTAGAAGTACCATTACTTAATACCTCTGTACTATTAGCTTCTGGAGTATCAATCACTTGAGCCCACCATAGCTTAATAGAAGGTAATCGAAAACACATCTTATACGACAACT
GAAATACCACTTTACATCAAATCATCATTTTGGATTTGAAGCCGCTGCTTGATATTGACATTTCGTAGATGTAGTGTGACTATTCCTTTACGTAATACTTTATTATCCTCACTCCTCGTTTTAAT
TGCATTTTGACTACCCCAATTAAATATTTACGCAGAAAAAGCAAGCCCCTATGAATGCGGATTTGACCCAATAGGATCTGCTCGTCTGCCTTTCTCTATAAATTTTTCTTACATTTCTACTATTT
GACCTAGAAATTGCACTACTATTACCCCTACCCTGAGCCTCACAAACAAATAACTTATAACAATACTTACTCTTTTAATTCGCTCTTGGCTGCAAGCCTAGCTTATGAATGAACCCAAAAAGGAC
TAGAATGATCAGAATATGATAATTATGCCTGTGAAGCAGCCCTGGTTAATATTATCCTCGAACTATAATTCTAGCACGAGGACTACAGACATCCTCCCTCTAATGGTAAATCACCCACCATATCA
AAAGCATCAAGCCATCCTTCACACGAGAAAACGCCCTAATAACCCTTCACCTGCTACCCCTTTTATTACTATCACTAAACCCCATAAATTCATGCTCCCATGTATGAAAACATGGCTTTTTCAAC
TTTTATAGGATAGAAGTAATCCATTGGCCTTAGGAGCCAAAAAATTGGTGCAACTCCAAATAAAAGTAATAAATTCATTCGCCTCCTTTATATTAACAGACCGCCAACAACCTATTCCAACTATT
TATCGGATGAGAAGGAGTGGGAATTATATCCTTTCTACTAATTGGCTGATGATATGGCCGAACTGACGCAAATACTGCCGCTCTACAGGCCATCTTATATAACCGTATCGATAGCCATAGATGTT
TCTTACTAACCTAAACGAATCAACCTTATCTCGCATTTCTGCACATCTGCACGCATGCATTCTTCAAAGCCATATTATTTATATGCTCAGGATCAATTATCCACAGCCTAAATGACGAGCAAGAT
ATCCGAAAAATAGGCGGACTTTATAAATCTATACCATTTACCACCACCTCCCTAATTATCGGAAGCCTCGCACTCACAGGAATACCTTTCCTAACAGGCTTTTACTCCAAAGACCTAATTATCGA
GACCGCCAATACGTCGTATACCAACGCCTGAGCCCTATTAACTACTCTCATCGCCACATCCCTGACAGCTGCCTACAGCACTCGAATTATATTTTTTGCACTTCTAGGACAACCACGCTTCAACC
TTAATATGAGCCAAAAATCCGCATCAATACTACTAGACATAATCTGATTAAAAATACCTCCTCACTAAAAAATCCTGAATCTCCCGTATCATAAATTACTCAATCACCCGCACCATTAAATTTAA
ACACAATCTCAACCCATCCCCTTTAAAATATAGATTAAAAAAACCATTAACCCTAAAAAGGACCCCCCAAAGTTCAATACAATACCACAACCAACTCCACCAGCTACAATCAAACCAAACCCACC
ATAAATAGGTGAAGGCTTTGAAGAAAAACTCACAAAGCTAACCACAAAAATAGTACTTAAAATGAATACAATGTATGTCATCATTATTCTCACATGGAATCTAACCATGACTAATGATATGAAAA
ACCATCGTTGTATTTCAACTACAAGAATTAATGACCAACATCCGAAAATCCCACCCCTAATAAAATCATCAACGAATCCTTTATTGATCTTCCAACTCCACTATATCTCAGCATGATGAAACTTT
GGTTCCCTATTAGGAATCTGCCTAGTTCTACAAATCTTAACAGGCTTATTTCTAGCCATACACTACACATCAGACACAGCAACCGCCTTCTCATCAGTAACCCATATTTGCCGCGACGTCAACTA
CGGATGAATTATCCGATATATACATGCTAATGGAGCACTGATCTGGTAGAATGAATCTGAGGCGGCTTCTCAGTAGACAAGGCCACCTTGACACGGTTTTTCGCCTTTCACTTCATCCTTCCATT
CATCATCGCCGCTCTAGCAGCAGTCCACCTCCTATTCCTTCATGAAACTGGATCAAACAACCCCTCAGGTAAATCCCCACCCCATATCAAACCCGAATGGTATTTTCTATTCGCATACGCAATTC
TCCGATCCATCCCTAATAAATTAGGAGGAATCTCCTAAAGTGAAGAGTCTTTGTAGTATATAAAATACCTTGGTCTTGTAAACCAAAAAAGGAGAACATGATCCCTCCCTAAGACTTCAAGGAAG
AAGCAACTGCCCCACCACCAGCACCCAAAGCTGAAATTCTTTTTAAACTATTCCTTGCTAATACCAAAAATCAGCTCTCCAACATTCATAATTCATATATTGCATGA
>PLI-5.sort.StEndRmdup_3x-90p
TCCAAGTTAATGTAGCTTAAATCATAAAGCAAGGCACTGAAAATGCCTAGATGAGTTGTAATAACTCCATAAACACAAAGGTTTGGTCCTGGCCTTTCTATTAGTTATTAGTAGGATTACACATG
CAAGCCTCCGCATCCCGGTGAAAATACCCTCTATGTCATTTATGACCCAAAGGAGTAGACATCAAGCACACAACTACTGTAGCTCATGACGTCTTGCAAAGCCACACCCCCACGGGATACAGCAG
TGATAAAAATTAAGCTATGAATGAAAGTTCGACTTAGCCATACTAAACTTAAGGGTTGGTAAATTTCGTGCCAGCCACCGCGGTCATACGATTAACCCGAACTAATAGATCTACGGCGTAAAGCG
TGTTAAAGAAATAAGCCTCACTAGAGTTAAGTCTTAACTAACAGAAAGTAGTCTAATATCTCTAACTACACGAAAGCTAAGATCCAAACTGGGATTAGATACCCCACTATGCTTAGCCCTAAACC
TAGATGACTTTATCCAACAAAGTTATCCGCCAGAGAACTACTAGCAACAGCTTAAAACTCAAAGGACTTGGCGGTGCTTTATATCCCTCTAGAGGAGCCTGTTCTATAATCGATAAACCCCGATA
TACCTCACCACTCCTTGCTAATCCAGTTTATATACCGCCATCTTCAGCAAACCCTTAAAAGGAATAAAAGTAAGCACAAGTATTTTTAACACAAAAAAGTTAGGTCAAGGTGTAACCTATGGAGT
GGGAAGTAATGGGCTACATTTTCTGATTATGAAATTAAGAACTAAAGGAGGATTTAGCAGTAAATTGAGAATAGAGTGCTCAATTGAATCGGGCCATGAAGCACGCACACACCGCCCGTCACCCT
CCTCAAGTGACAATCTATAAATAACATAATTACCCCATAAAATCACAAGAGGAGACAAGTCGTAACAAGGTAAGCATACTGGAAAGTGTGCTTGGATAACCAAGATGTAGCTTAAGTAAAGCATC
TGGCCTACACCCAGAAGATTTCATATTAAACTGAACATCCTGAGCTAAAACTAGCCCAAACAACTACAAACTCAACTTACACTTAACAATAAATCAACCATTTAATTACTAAAATTAAAAGTATA
GGAGATAGAAATTTAACTTGGCGCCATAGAGATAGTACCGCAAGGGAAAGATGAAAGAAATATTTAAAGCACCACACAGCAAAGATTATCCCTTGTACCTTTTGCATAATGAATCAGCTAGAACG
CCCTAGCAAAGAGAACTTAAGCTAGACTCCCCGAAACCAGACGAGCTACCTGTGAACAATCTACATGGGATGAACTCATCTATGTCGCAAAATAGTGAGAAGATTTACAGGTAGAGGTGAAAAGC
CTAACGAGCCTGGTGATAGCTGGTTGCCCAGGATAGAATTTTAGTTCAACTTTAAACTTACCCGCAAATCTCCCTTAGAATTATAATGTAAGTTTAAAATATAATCTAAAAAGGTACAGCTTTTT
AGAACTAGATACAGCCTTTTTTAGTGAGTAAGTATAATTATTAACCATAGTCGGCCTAAAAGCAGCCATCAATTAAGAAAGCGTTCAAGCTCAACAGTTAAAATATCTTAATATCAAAAATAATT
ATATAATAGAAGCAATAATGCTGATATGAGTAACAAGAAATATTTCTCCTTGCATAAGCTTATATCAGAGCGGATAACCACTGATAGTTAACAACAAGATATGAAAAACCCACCTATAAATTCAA
TATCAAGTTAATTGTTACCCAACACAGGCATGCAATCAAGGAAAGATTAAAAGAAGTAAAAGGAACTCGGCAAACACAAGCCCCGCCTGTTTACCAAAAACATCACCTCTAGCATTTCTAGTATT
AGAGGCACTGCCTGCCCAGTGACATTAGTTAAACGGCCGCGGTATCCTGACCGTGCAAAGGTAGCATAATCACTTGTTCCCTAAATAGGGACTCGTATGAATGGCCACACGAGGGCTTTACTGTC
TCTTACTTCCAATCCGTGAAATTGACCTTCCCGTGAAGAGGCGGGAATGTGACAATAAGACGAGAAGACCCTATGGAGCTTTAATTAACTAACTCAGAGAGATTACTTTAACATACGATAACAAA
CCTCTACATGAGTTAGCAATTGGGTTGGGGTGACCTCGGAGAACAAAAAAACCTCCGAGTGATTTTAATCTAGACTAACCAGTCAAAAGTATTACATCACTTATTGATCCAAAAACTTGATCAAC
```

GGAACAAGTTACCCTAGGGATAACAGCGCAATCCTATTTTAGAGTCCATATCGACAATAGGGTTTACGACCTCGATGTTGGATCAGGACATCCCGATGGTGCAGCAGCTATCAAAGGTTCGTTTG
TTCAACGATTAAAGTCCTACGTGATCTGAGTTCAGACCGGAGTAATCCAGGTCGGTTTCTATCTATTAAACAATTTCTCCCAGTACGAAAGGACAAGAGAAATAGGGCCTACCTTACTAAGACGC
CCTCAACTGTAGACATACGGGTTCGTTAGGGTGGCAGAGCCCGGTAATTGCATAAAACTTAAACTTTTACAATCAGAGGTTCAATTCCTCTCCCTAACAATATGTTCATAATTAATATTCTATCA
CTAATCGTTCCAATCCTCCTCGCCGTAGCCTTCCTAACACTAGTTGAACGTAAAGTCTTAGGTTACATACAACTCCGCAAGGGACCAAACATTGTAGGACCATACGGTCTGCTCCAACCCATCGC
AGACGCCATTAAATTATTTACCAAAGAACCCCTGCGACCCCTAACATCCTCCATATCAATATTCATCATAGCACCAATCCTAGCCCTTCACTGCCCTAACCATATGAATCATAAACTTCATACTA
GCCATATAAGTTTAGCCGTATATTCCATTTTATGCCTCAAACTCCAAATATGCCTTAATTGGAGCTCTACGAGCTGTAGCCCAAACAATCTCATATGAAGTCACACTAGCTATTATTCTCCTATC
CGTGCTCTAATTACCACCCAAGAATATATATGGCTAATCTTTCCCGCATGACCCCTAGCCATAATATGATTTATCTCAACCCTAGCAGAAACCAACCGAGCTCCATTCGACCTTACAGAAGGAGA
GTCAGAACTCGTCTCTGGCTTCAACTAGAATTTATTCCTAACTATCTCCTTCCGAATTCGAGCATCATACCCCCGATTCCGATATGATCAACTTATACACCTACTATGAAAAAACTTCCTACCCC
TAACTCTAGCCCTATGCATATGACACGTATCCCTTCCCATTATAACTGCAAGTATCCCACCTCAAACATAAGAAATATGTCTGACAAAAGAGTTACTTTGATAGAGTAAACAATAGAGGTTTAAG
CCCTCTTATTTCTAGAGCTATAGGAATCGAACCTAATCCTAAGAATTCAAAAATCTTCGTGCTACCATATTACACCACGCTCTATAGTAAGGTCAGCTAAGTAAGCTATCGGGCCCATACCCCGA
AAATGTTGGTTTATACCCTTCCCGTACTAATTAAACCGCCTATTCTCATCATTATTATATCAACTGTAATCTCAGGGAAATTGTAAAGCTCTAACAATCTGAATCGGCTTTGAAATAAATATTCT
TATAAAAAAATTTAACCCCCGAGCTATAGAGGCAGCTACAAAATATTTCCTCACACAAGCAACCGCATCCATACTATTAATAATAGGAATTATCATCAAATAAACCCTCACCCCATAGCATCAAT
TATAATAACCACAGCCCTAGCAATAAAACTAGGTCTAGCCCCATTCCACTTCTGAGTCCCCGAAGTAGCACAAGGAATTTCTCTATCCTCAAGTCTCATTTTATTAACATGACAAAAATACTTCT
AACAATAGCTATTACATCAGTTATCATCGGGGGCTGAGGAGGACTAAATCAAACCCAACTACGAAAAATCATAGCATATTCCTCAATTGCACACATATTCATACATAGCTCTTCTACCACAACAT
ATCCCTATCCCAAACATAAACAAAGCACCCTTGATCACCTCACTACCATTATCCCCACATACTAGCCATCACAGCACTACTCAATCTATACTTCTACATACGACTAACGTACGCCACAGCACTTA
CTATATTTCCATCAGCTAAAACATAAAAATAACACTTTCAACTATACTCTCCCCCTGCCCCCATAATATCCATCCTGGATTAGAAGTTTAGGTTAGACAGACCAAGAGCCTTCAAAGCCCTAAGC
AAGCCTCACTGGCTTAACTTCTGAACTCCCATAAGGACTGCAAGAATCTATCTCACATCAATTGATTGCAAATCAAACACTTTAATTAAGCTAAGCCCTTACTAGATTGGTGGGATCCAACCCCA
CGAAACTTTAGTTAACAGCTAAATACCCTAATCAACTGGCTTCAATCTACTTCTCCCGCCGTCTAGAAAAAAAAGGCGGGAGAAGCCCCGGCAGCGTTAAAGCTGCTTCTTTGAATTTGCAATTC
AATATGAATATTCACCACAGGACTTGGTAAAAAGAGGATTAAACCTCTGTTCTTAGATTTACAGTCTAATGCTTTGCTCAGCCATTTTACCTATGTTCATAAACCGTTGACTATTTTCTACTAAC
CACAAAGATATCGGTACTCTATTCAATAGTAGAAGCCGGGGCAGGAACCGGATGAACAGTTCTAGAGGTAATTTAGCACATGCAGGAGCATCGTAGACACCACAATCATTAACATAAAACCTCCT
GCCATATCCCAATACCAAACACCTTTATTTGTCTGATCAGTCTTAATCACGCTGTTCTATTCGTGTGAGCTCACCATATGTTTACTGTAGGCATAGATGTGGACACACGAGCATATTTTACATCA
GCTACTATAATTATCGCAATTATGTAGTAGCCCATTTCCACTATGTATTATCAATAGGAGCAGTTTTCGCTATTATAGGAGGCTTTGTACATTGATTTCCTTTATTTGAAGAACTGCCTACGTAT
CTCTAAAGTAAGAAAGGAAGGAATCGAACCTCCTAGGACTGGTTTCAAGCCAATATCATAACCATTACACTCTGAGCGGTCCATCACTAGGACTATAAATCAAACAACTTTAATAGGCACACGCC
CAGGGTTATACTATGGTCAATGCTCAGAAATCTGTGGCTCAAACCATAGTTTTATACCTATCGTCCTTGAATTAGTACCATTAACATATTTCGAGAAACCCCTGAAGTCCAGAACCTAAATCTGC
AATTACACTAAAACAACCTAATCCTTGAGAAAAAAAATGAACGAAAATCTATTCGCCTCTTTCACTACCCCAACGATAATAGGCTTGCCAATTGTTATCTTAATCGTATTATTATCCTAGTCCTA
CTAACAATCCTTGAATTCGCAGTAGCCCTAATTCAAGCCTATGTCTTTACCCTGCTAGTAAGCCTATACTTACATGATCATATATATACCAATGATGACGAGATGTTATTCGAGAAAGCACATTT
CAAGGCCACCACACACCCGTTGTTCAAAAAGGATTGCGATATGGAATAATCCTATTTATTGATCTGAAGCTGAGCATTTTATCACTCAAGCTCCAGATTAGGAGGTTGCTGACCCACAGGCATTA
TTCCTCTAATCCTCTAGAAGTACCATTACTTAATACCTCTGTACTATTAGCTTCTGGAGTATCAATCACTTGAGCCCACCATAGCTTAATAGAAGGTAATCGAAAGCACATCTTATACGACAACT
GAAATACCACTTTACATCAAATCATCATTTTGGATTTGAAGCCGCTGCTTGATATTGACATTTCGTAGATGTAGTGTGACTATTCCTTTACGTAATACTTTATTATCCTCACTCCTCGTTTTAAT
TGCATTTTGATTACCCCAATTAAATATTTACGCAGAAAAAGCAAGCCCCTATGAATGCGGATTTGACCCAATAGGATCTGCTCGTCTGCCTTTCTCTATAAATTTTTCTTACATTTCTACTATTT
GATCTAGAAATTGCACTACTATTACCCCTGCCCTGAGCCTCACAAACAAATAACTTATAACAATACTTACTCTCTTAATTCGCTCTTGGCTGCAAGCCTAGCTTATGAATGAACCCAAAAAGGAC
TAGAATGATCAGAATATGATAATTATGCCTGTGAGGCAGCCCTGGTTAATATTATCTTCGAACTATAATTCTAGCACGAGGACTACAGACATCCTTCCCCTAATGGTAAATTACCCACCATATCA
AAAGCATCAAGCCATCCTTCACACGAGAAAACGCCCTAATAACCCTTCACCTGCTACCCCTTTTATTACTATCACTAAACCCTATAAATTCATGCTCCCATGTATGAAAACATGGCTTTTTCAAC
TTTTATAGGATAGAAGTAATCCATTGGCCTTAGGAGCCAAAAAATTGGTGCAACTCCAAATAAAAGTAATAAATCCATTCGCCTCCTTTATATTAACAGACCGCCAACAACCTATTCCAACTATT
TATCGGATGAGAAGGGGTAGGAATTATATCCTTTCTACTAATTGGCTGATGATATGGCCGAACTGACGCAAATACTGCCGCTCTACAGGCCATTTTATATAACCGTATTGATAGCCATAGATGTT
TCTTACTAACCTAAACGAATCAACCTTATCTCGCATTTCTGCACATCTGCACGCATGCATTCTTCAAAGCCATATTATTTATATGCTCAGGATCAATTATCCACAGCCTAAATGATGAACAAGAT
ATCCGAAAAATAGGCGGACTTTATAAATCTATACCATTTACCACCACCTCCCTAATTATCGGAAGCCTCGCACTCACAGGAATACCCTTCCTAACAGGCTTCTACTCCAAAGACCTAATTATCGA
GACCGCCAATACGTCGTATACCAACGCCTGAGCCCTATTAACTACTCTCATCGCCACATCCCTGACAGCTGCCTACAGCACTCGAATTATATTTTTTGCACTTCTAGGACAACCACGCTTCAACC
TTAATATGAGCCAAAAATCCGCATCAATACTACTAGACATAATCTGATTAAAAATACCTCCTCACTAAAAAATCCTGAATCTCCCGTATCATAAATCACTCAATCACCCGCACCATTAAATTTAA
ACACAATCTCAACCCATCCCCTTTAAAATATAGATTAAAAAAACTATTAATCCTAAAAAGGATCCCCCAAAGTTCAATACAATACCACAACCAACTCCACCAGCTACAATCAAACCAAACCCACC
ATAAATAGGTGAAGGCTTTGAAGAAAAACTCACAAAGCTAACCACAAAAATAGTACTTAAAATGAATACAATGTATGTCATCATTATTCTCACATGGAATCTAACCATGACTAATGATATGAAAA
ACCATCGTTGTATTTCAACTACAAGAATTAATGACCAACATCCGAAAATCCCACCCCTAATAAAATCATCAACGAATCCTTTATTGATCTTCCAACTCCACTACATCTCAGCATGATGAAACTTT
GGTTCCCTATTAGGAATCTGCCTGATTCTACAAATCTTAACAGGCTTATTTCTAGCCATACACTACACATCAGACACAGCAACCGCCTTCTCATCAGTAACCCATATTTGCCGCGACGTCAACTA
CGGATGAATCATCCGATACATACATGCTAATGGAGCACTGATCTAGTAGAATGAATTTGAGGCGGCTTCTCAGTAGACAAGGCCACCTTGACACGGTTTTTCGCCTTTCACTTCATCCTTCCATT
CATCATCGCCGCTCTAGCAGCAGTCCACCTCCTATTCCTTCATGAAACTGGATCAAACAACCCCTCAGGTAAATCCCCACCCCATATCAAACCCGAATGGTATTTTCTATTCGCATACGCAATCC
TCCGATCCATCCCTAATAAATTAGGAGGAATCTCCTAAAGTGAAGAGTCTTTGTAGTATATAAAATACCTTGGTCTTGTAAACCAAAAAAGGAGAACATAATCCCTCCCTAAGACTTCAAGGAAG
AAGCAACTGCCCCACCACCAGCACCCAAAGCTGAAATTCTTTTTAAACTATTCCTTGCTAATACCAAAAATCAGCCCTCCAACATTCATAATTCATATATTGCATGA
>PLI-20_merged.sort.StEndRmdup_2_
TCCAAGTTAATGTAGCTTAAATCATAAAGCAAGGCACTGAAAATGCCTAGATGAGTTGTAATAACTCCATAAACACAAAGGTTTGGTCCTGGCCTTTCTATTAGTTATTAGTAGGATTACACATG
CAAGCCTCCGCATCCCGGTGAAAATACCCTCTATGTCATTTATGACCCAAAGGAGTAGACATCAAGCACACAACTACTGTAGCTCATGACGTCTTGCAAAGCCACACCCCCACGGGATACAGCAG
TGATAAAAATTAAGCTATGAATGAAAGTTCGACTTAGCCATACTAAACTTAAGGGTTGGTAAATTTCGTGCCAGCCACCGCGGTCATACGATTAACCCGAACTAATAGATCTACGGCGTAAAGCG
TGTTAAAGAAATAAGCCTCACTAGAGTTAAGTCTTAACTAACAGAAAGTAGTCTAATATCTCTAACTACACGAAAGCTAAGATCCAAACTGGGATTAGATACCCCACTATGCTTAGCCCTAAACC
TAGATGACTTTATCCAACAAAGTTATCCGCCAGAGAACTACTAGCAACAGCTTAAAACTCAAAGGACTTGGCGGTGCTTTATATCCCTCTAGAGGAGCCTGTTCTATAATCGATAAACCCCGATA
TACCTCACCACTCCTTGCTAATCCAGTTTATATACCGCCATCTTCAGCAAACCCTTAAAAGGAATAAAAGTAAGCACAAGTATTTTTAACACAAAAAGTTAGGTCAAGGTGTAACCTATGGAGT
GGGAAGTAATGGGCTACATTTTCTGATTATGAAATTAAGAACTAAAGGAGGATTTAGCAGTAAATTGAGAATAGAGTGCTCAATTGAATCGGGCCATGAAGCACGCACACACCGCCCGTCACCCT
CCTCAAGTGACAATCTATAAATAACATAATTACCCCATAAAATCACAAGAGGAGACAAGTCGTAACAAGGTAAGCATACTGGAAAGTGTGCTTGGATAACCAAGATGTAGCTTAAGTAAAGCATC
TGGCCTACACCCAGAAGATTTCATATTAAACTGAACATCCTGAGCTAAAACTAGCCCAAACAACTACAAACTCAACTTACACTTAACAATAAATCAACCATTTAATTACTAAAATTAAAAGTATA
GGAGATAGAAATTTAACTTGGCGCCATAGAGATAGTACCGCAAGGGAAAGATGAAAGAAATATTTAAAGCACCACACAGCAAAGATTATCCCTTGTACCTTTTGCATAATGAATCAGCTAGAACG
CCCTAGCAAAGAGAACTTAAGCTAGACTCCCCGAAACCAGACGAGCTACCTGTGAACAATCTACATGGGATGAACTCATCTATGTCGCAAAATAGTGAGAAGATTTACAGGTAGAGGTGAAAAGC
CTAACGAGCCTGGTGATAGCTGGTTGCCCAGGATAGAATTTTAGTTCAACTTTAAACTTACCCGCAAATCTCCCTTAGAATTATAATGTAAGTTTAAAATATAATCTAAAAGGTACAGCTTTTT
AGAACTAGATACAGCCTTTTTTAGTGAGTAAGTATAATTATTAACCATAGTCGGCCTAAAAGCAGCCATCAATTAAGAAAGCGTTCAAGCTCAACAGTTAAAATATCTTAATATCAAAAATAATT
ATATAATAGAAGCAATAATGCTGATATGAGTAACAAGAAATATTTCTCCTTGCATAAGCTTATATCAGAGCGGATAACCACTGATAGTTAACAACAAGATATGAAAAACCCACCTATAAATTCAA
TATCAAGTTAATTGTTACCCAACACAGGCATGCAATCAAGGAAAGATTAAAAGAAGTAAAAGGAACTCGGCAAACACAAGCCCCGCCTGTTTACCAAAAACATCACCTCTAGCATTTCTAGTATT
AGAGGCACTGCCTGCCCAGTGACATTAGTTAAACGGCCGCGGTATCCTGACCGTGCAAAGGTAGCATAATCACTTGTTCCCTAAATAGGGACTCGTATGAATGGCCACACGAGGGCTTTACTGTC
TCTTACTTCCAATCCGTGAAATTGACCTTCCCGTGAAGAGGCGGGAATGTGACAATAAGACGAGAAGACCCTATGGAGCTTTAATTAACTAACTCAGAGAGATTACTTTAACATACGATAACAAA
CCTCTACATGAGTTAGCAATTGGGTTGGGGTGACCTCGGAGAACAAAAAAACCTCCGAGTGATTTTAATCTAGACTAACCAGTCAAAAGTATTACATCACTTATTGATCCAAAAACTTGATCAAC
GGAACAAGTTACCCTAGGGATAACAGCGCAATCCTATTTTAGAGTCCATATCGACAATAGGGTTTACGACCTCGATGTTGGATCAGGACATCCCGATGGTGCAGCAGCTATCAAAGGTTCGTTTG
TTCAACGATTAAAGTCCTACGTGATCTGAGTTCAGACCGGAGTAATCCAGGTCGGTTTCTATCTATTAAACAATTTCTCCCAGTACGAAAGGACAAGAGAAATAGGGCCTACCTTACTAAGACGC
CCTCAACTGTAGACATACGGGTTCGTTAGGGTGGCAGAGCCCGGTAATTGCATAAAACTTAAACTTTTACAATCAGAGGTTCAATTCCTCTCCCTAACAATATGTTCATAATTAATATTCTATCA
CTAATCGTTCCAATCCTCCTCGCCGTAGCCTTCCTAACACTAGTTGAACGTAAAGTCTTAGGTTACATACAACTCCGCAAGGGACCAAACATTGTAGGACCATACGGTCTGCTCCAACCCATCGC
AGACGCCATTAAATTATTTACCAAAGAACCCCTGCGACCCCTAACATCCTCCATATCAATATTCATCATAGCACCAATCCTAGCCCTTCACTGCCCTAACCATATGAATCATAAACTTCATACTA
GCCATATAAGTTTAGCCGTATATTCCATTTTATGCCTCAAACTCCAAATATGCCTTAATTGGAGCTCTACGAGCTGTAGCCCAAACAATCTCATATGAAGTCACACTAGCTATTATTCTCCTATC
CGTGCTCTAATTACCACCCAAGAATATATATGGCTAATCTTTCCCGCATGACCCCTAGCCATAATATGATTTATCTCAACCCTAGCAGAAACCAACCGAGCTCCATTCGACCTTACAGAAGGAGA
GTCAGAACTCGTCTCTGGCTTCAACTAGAATTTATTCCTAACTATCTCCTTCCGAATTCGAGCATCATACCCCCGATTCCGATATGATCAACTTATACACCTACTATGAAAAAACTTCCTACCCC
TAACTCTAGCCCTATGCATATGACACGTATCCCTTCCCATTATAACTGCAAGTATCCCACCTCAAACATAAGAAATATGTCTGACAAAAGAGTTACTTTGATAGAGTAAACAATAGAGGTTTAAG
CCCTCTTATTTCTAGAGCTATAGGAATCGAACCTAATCCTAAGAATTCAAAAATCTTCGTGCTACCATATTACACCACGCTCTATAGTAAGGTCAGCTAAGTAAGCTATCGGGCCCATACCCCGA
AAATGTTGGTTTATACCCTTCCCGTACTAATTAAACCGCCTATTCTCATCATTATTATATCAACTGTAATCTCAGGGAAATTGTAAAGCTCTAACAATCTGAATCGGCTTTGAAATAAATATTCT
TATAAAAAAATTTAACCCCCGAGCTATAGAGGCAGCTACAAAATATTTCCTCACACAAGCAACCGCATCCATACTATTAATAATAGGAATTATCATCAAATAAACCCTCACCCCATAGCATCAAT
TATAATAACCACAGCCCTAGCAATAAAACTAGGTCTAGCCCCATTCCACTTCTGAGTCCCCGAAGTAGCACAAGGAATTTCTCTATCCTCAAGTCTCATTTTATTAACATGACAAAAATACTTCT
AACAATAGCTATTACATCAGTTATCATCGGGGGCTGAGGAGGACTAAATCAAACCCAACTACGAAAAATCATAGCATATTCCTCAATTGCACACATATTCATACATAGCTCTTCTACCACAACAT
ATCCCTATCCCAAACATAAACAAAGCACCCTTGATCACCTCACTACTATTATCCCCACATACTAGCCATCACAGCACTACTCAATCTATACTTCTACATACGACTAACGTACGCCACAGCACTTA
CTATATTTCCATCAGCTAAAACATAAAAATAACACTTTCAACTATACTCTCCCCCTGCCCCCATAATATCCATCCTGGATTAGAAGTTTAGGTTAGACAGACCAAGAGCCTTCAAAGCCCTAAGC
AAGCCTCACTGGCTTAACTTCTGAACTCCCATAAGGACTGCAAGAATCTATCTCACATCAATTGATTGCAAATCAAACACTTTAATTAAGCTAAGCCCTTACTAGATTGGTGGGATCCAACCCCA
CGAAACTTTAGTTAACAGCTAAATACCCTAATCAACTGGCTTCAATCTACTTCTCCCGCCGTCTAGAAAAAAAAGGCGGGAGAAGCCCCGGCAGCGTTAAAGCTGCTTCTTTGAATTTGCAATTC
AATATGAATATTCACCACAGGACTTGGTAAAAAGAGGATTAAACCTCTGTTCTTAGATTTACAGTCTAATGCTTTGCTCAGCCATTTTACCTATGTTCATAAACCGTTGACTATTTTCTACTAAC
CACAAAGATATCGGTACTCTATTCAATAGTAGAAGCCGGGGCAGGAACCGGATGAACAGTTCTAGAGGTAATTTAGCACATGCAGGAGCATCGTAGACACCACAATCATTAACATAAAACCTCCT
GCCATATCCCAATACCAAACACCTTTATTTGTCTGATCAGTCTTAATCACGCTGTTCTATTCGTGTGAGCTCACCATATGTTTACTGTAGGCATAGATGTGGACACACGAGCATATTTTACATCA
GCTACTATAATTATCGCAATTATGTAGTAGCCCATTTCCACTATGTATTATCAATAGGAGCAGTTTTCGCTATTATAGGAGGCTTTGTACATTGATTTCCTTTATTTGAAGAACTGCCTACGTAT
CTCTAAAGTAAGAAAGGAAGGAATCGAACCTCCTAGGACTGGTTTCAAGCCAATATCATAACCATTACACTCTGAGCGGTCCATCACTAGGACTATAAATCAAACAACTTTAATAGGCACACGCC

```
CAGGGTTATACTATGGTCAATGCTCAGAAATCTGTGGCTCAAACCATAGTTTTATACCTATCGTCCTTGAATTAGTACCATTAACATATTTCGAGAAACCCCTGAAGTCCAGAACCTAAATCTGC
AATTACACTAAAACAACCTAATCCTTGAGAAAAAAAATGAACGAAAATCTATTCGCCTCTTTCACTACCCCAACGATAATAGGCTTGCCAATTGTTATCTTAATCGTATTATTATCCTAGTCCTA
CTAACAATCCTTGAATTCGCAGTAGCCCTAATTCAAGCCTATGTCTTTACCCTGCTAGTAAGCCTATACTTACATGATCATATATATACCAATGATGACGAGATGTTATTCGAGAAAGCACATTT
CAAGGCCACCACACACCCGTTGTTCAAAAAGGATTGCGATATGGAATAATCCTATTTATTGATCTGAAGCTGAGCATTTTATCACTCAAGCTCCAGATTAGGAGGTTGCTGACCCACAGGCATTA
TTCCTCTAATCCTCTAGAAGTACCATTACTTAATACCTCTGTACTATTAGCTTCTGGAGTATCAATCACTTGAGCCCACCATAGCTTAATAGAAGGTAATCGAAAGCACATCTTATACGACAACT
GAAATACCACTTTACATCAAATCATCATTTTGGATTTGAAGCCGCTGCTTGATATTGACATTTCGTAGATGTAGTGTGACTATTCCTTTACGTAATACTTTATTATCCTCACTCCTCGTTTTAAT
TGCATTTTGATTACCCCAATTAAATATTTACGCAGAAAAAGCAAGCCCCTATGAATGCGGATTTGACCCAATAGGATCTGCTCGTCTGCCTTTCTCTATAAATTTTTCTTACATTTCTACTATTT
GATCTAGAAATTGCACTACTATTACCCCTGCCCTGAGCCTCACAAACAAATAACTTATAACAATACTTACTCTCTTAATTCGCTCTTGGCTGCAAGCCTAGCTTATGAATGAACCCAAAAAGGAC
TAGAATGATCAGAATATGATAATTATGCCTGTGAGGCAGCCCTGGTTAATATTATCTTCGAACTATAATTCTAGCACGAGGACTACAGACATCCTTCCCCTAATGGTAAATTACCCACCATATCA
AAAGCATCAAGCCATCCTTCACACGAGAAAACGCCCTAATAACCCTTCACCTGCTACCCCTTTTATTACTATCACTAAACCCTATAAATTCATGCTCCCATGTATGAAAACATGGCTTTTTCAAC
TTTTATAGGATAGAAGTAATCCATTGGCCTTAGGAGCCAAAAAATTGGTGCAACTCCAAATAAAAGTAATAAATCCATTCGCCTCCTTTATATTAACAGACCGCCAACAACCTATTCCAACTATT
TATCGGATGAGAAGGGGTAGGAATTATATCCTTTCTACTAATTGGCTGATGATATGGCCGAACTGACGCAAATACTGCCGCTCTACAGGCCATTTTATATAACCGTATTGATAGCCATAGATGTT
TCTTACTAACCTAAACGAATCAACCTTATCTCGCATTTCTGCACATCTGCACGCATGCATTCTTCAAAGCCATATTATTTATATGCTCAGGATCAATTATCCACAGCCTAAATGATGAACAAGAT
ATCCGAAAAATAGGCGGACTTTATAAATCTATACCATTTACCACCACCTCCCTAATTATCGGAAGCCTCGCACTCACAGGAATACCCTTCCTAACAGGCTTCTACTCCAAAGACCTAATTATCGA
GACCGCCAATACGTCGTATACCAACGCCTGAGCCCTATTAACTACTCTCATCGCCACATCCCTGACAGCTGCCTACAGCACTCGAATTATATTTTTTGCACTTCTAGGACAACCACGCTTCAACC
TTAATATGAGCCAAAAATCCGCATCAATACTACTAGACATAATCTGATTAAAAATACCTCCTCACTAAAAAATCCTGAATCTCCCGTATCATAAATCACTCAATCACCCGCACCATTAAATTTAA
ACACAATCTCAACCCATCCCCTTTAAAATATAGATTAAAAAAACTATTAATCCTAAAAAGGATCCCCCAAAGTTCAATACAATACCACAACCAACTCCACCAGCTACAATCAAACCAAACCCACC
ATAAATAGGTGAAGGCTTTGAAGAAAAACTCACAAAGCTAACCACAAAAATAGTACTTAAAATGAATACAATGTATGTCATCATTATTCTCACATGGAATCTAACCATGACTAATGATATGAAAA
ACCATCGTTGTATTTCAACTACAAGAATTAATGACCAACATCCGAAAATCCCACCCCTAATAAAATCATCAACGAATCCTTTATTGATCTTCCAACTCCACTACATCTCAGCATGATGAAACTTT
GGTTCCCTATTAGGAATCTGCCTGATTCTACAAATCTTAACAGGCTTATTTCTAGCCATACACTACACATCAGACACAGCAACCGCCTTCTCATCAGTAACCCATATTTGCCGCGACGTCAACTA
CGGATGAATCATCCGATACATACATGCTAATGGAGCACTGATCTAGTAGAATGAATTTGAGGCGGCTTCTCAGTAGACAAGGCCACCTTGACACGGTTTTTCGCCTTTCACTTCATCCTTCCATT
CATCATCGCCGCTCTAGCAGCAGTCCACCTCCTATTCCTTCATGAAACTGGATCAAACAACCCCTCAGGTAAATCCCCACCCCATATCAAACCCGAATGGTATTTTCTATTCGCATACGCAATCC
TCCGATCCATCCCTAATAAATTAGGAGGAATCTCCTAAAGTGAAGAGTCTTTGTAGTATATAAAATACCTTGGTCTTGTAAACCAAAAAAGGAGAACATAATCCCTCCCTAAGACTTCAAGGAAG
AAGCAACTGCCCCACCACCAGCACCCAAAGCTGAAATTCTTTTTAAACTATTCCTTGCTAATACCAAAAATCAGCCCTCCAACATTCATAATTCATATATTGCATGA
>PLI-15.sort.StEndRmdup_3x-90p
TCCAAGTTAATGTAGCTTAAACCATAAAGCAAGGCACTGAAAATGCCTAGATGAGTTGTAATAACTCCATAGACATAAAGGTTTGGTCCTGGCCTTTCTATTAGTTATTAGTAGGATTACACATG
CAAGCCTCCGCATCCCGGTGAAAATACCCTCTATGTCATTCATGACCCAAAGGAGTAGACATCAAGCACACAACCACTGTAGCTCATGACGTCTTGCAAAGCCACACCCCCACGGGATACAGCAG
TGATAAAAATTAAGCTATGAATGAAAGTTCGACTAAGCTATACTAAACTTAAGGGTTGGTAAATTTCGTGCCAGCCACCGCGGTCATACGATTAACCCGAACTAATAGATCTACGGCGTAAAGCG
TGTTAAAGAAATAAGCCTCACTAGAGTTAAGTCTTAACTAACAGAAAGTAGTCTAATATCTCTAACTACACGATAGCTAAGATCCAAACTGGGATTAGATACCCCACTATGCTTAGCCCTAAACC
TAGATGACTTTACCCAACAAAGTTATCCGCCAGAGAACTACTAGCAACAGCTTAAAACTCAAAGGACTTGGCGGTGCTTTATATCCCTCTAGAGGAGCCTGTTCTATAATCGATAAACCCCGATA
TACCTCACCACTCCTTGCTAATCCAGTTTATATACCGCCATCTTCAGCAAACCCTTAAAAGGAATAAAAGTAAGCACAAGTATTTTTAACACAAAAAAGTTAGGTCAAGGTGTAACCTATGGAGT
GGGAAGTAATGGGCTACATTTTCTGATTATGAAATTAAGAACTAAAGGAGGATTTAGCAGTAAATTGAGAATAGAGTGCTCAATTGAATCGGGCCATGAAGCACGCACACACCGCCCGTCACCCT
CCTCAAGTGACAACCTATAAATAACATAATTATCCCATAAAATCACAAGAGGAGATAAGTCGTAACAAGGTAAGCATACTGGAAAGTGTGCTTGGATAACCAAGATGTAGCTTAAGTAAAGCATC
TGGCCTACACCCAGAAGATTTCATATTAAACTGAACATCCTGAGCTAAAACTAGCCCAAACAACTATAAACTCAACTTTCACTCAACAATAAATCAACCATTTAATTACTAAAATTAAAAGTATA
GGAGATAGAAATTTAACTTGGCGCCATAGAGATAGTACCGCAAGGGAAAGATGAAAGAAATATTTAAAGCACCACACAGCAAAGATTATCCCTTGTACCTTTTGCATAATGAATCAGCTAGAACG
CCCTAGCAAAGAGAACTTAAGCTAGACCCCCCGAAACCAGACGAGCTACCTGTGAACAATCTACATGGGATGAACTCATCTATGTCGCAAAATAGTGAGAAGATTTACAGGTAGAGGTGAAAAGC
CTAACGAGCCTGGTGATAGCTGGTTGCCCAGGATAGAATTTTAGTTCAACTTTAAACTTACCCGCAAATCTCCCTTAAAATTCTAATGTAAGTTTAAAATATAATCTAAAAAGGTACAGCTTTTT
AGAACTAGATACAGCCTTTTTTAGTGAGTAAGCATAATTATTAACCATAGTCGGCCTAAAAGCAGCCATCAATTAAGAAAGCGTTCAAGCTCAACAGTTAAAATATCTTAATATCAAAAGTAATT
ATATAATAGAAGCAATAATGCTGATATGAGTAACAAGAAATATTTCTCCTTGCATAAGCTTATATCAGAGCGGATAACCACTGATAGTTAACAACAAGATATGAAAAACCTACCTATGAATTCAA
TATCAAGTTAATTGTTACCCAACACAGGCATGCAATCAAGGAAAGATTAAAAGAAGTAAAAGGAACTCGGCAAACACAAGCCCCGCCTGTTTACCAAAAACATCACCTCTAGCATTTCTAGTATT
AGAGGCACTGCCTGCCCAGTGACATTAGTTAAACGGCCGCGGTATCCTGACCGTGCAAAGGTAGCATAATCACTTGTTCCCTAAATAGGGACTCGTATGAATGGCCACACGAGGGCTTTACTGTC
TCTTACTTCCAATCCGTGAAATTGACCTTCCCGTGAAGAGGCGGGAATGTGACAATAAGACGAGAAGACCCTATGGAGCTTTAATTAACTAACTCAGAGAGATTACTTTAACATACGATAACAAA
CCTCTATATGAGTTAGCAATTGGGTTGGGGTGACCTCGGAGAACAAAAAAACCTCCGAGTGATTTTAATCTAGACTAACCAGTCAAAAGTATTACATCACTTATTGATCCAAAAACTTGATCAAC
GGAACAAGTTACCCTAGGGATAACAGCGCAATCCTATTTTAGAGTCCATATCGACAATAGGGTTTACGACCTCGATGTTGGATCAGGACATCCCGATGGTGCAGCAGCTATCAAAGGTTCGTTTG
TTCAACGATTAAAGTCCTACGTGATCTGAGTTCAGACCGGAGTAATCCAGGTCGGTTTCTATCTATTAAACAATTTCTCCCAGTACGAAAGGACAAGAGAAATAGGGCCTACCTTACCAAGACGC
CCTCAACTGTAGATGTACGGGTTAGTTAGGGTGGCAGAGCCCGGTAATTGCATAAAACTTAAACTTTTACAATCAGAGGTTCAATTCCTCTCCCTAACAATATGTTCATAATTAATATTTTATCA
CTAATCGTTCCAATCCTTCTCGCCGTAGCCTTCCTAACACTAGTTGAACGCAAAGTCTTAGGTTACATACAACTCCGCAAGGGACCAAACATTGTAGGACCATACGGTCTGCTCCAACCCATCGC
AGACGCCATTAAATTATTTACCAAAGAACCCCTGCGACCCCTAACATCCTCCATATCAATATTCATCATAGCACCAATCCTAGCCCTTCACTGCTCTAACCATATGAATCATAAACTTCATACTA
GCCATATAAGTTTAGCCGTATATTCCATTTTATGCCTCAAACTCCAAATATGCCTTAATTGGAGCTCTACGAGCCGTAGCCCAAACAATCTCATATGAAGTCACACTAGCTATCATTCTCCTATC
CGTGCTCTAATTACCACCCAAGAATATATATGGCTAATCTTTCCCGCATGACCCCTAGCCATAATATGATTTATCTCAACTCTAGCAGAAACCAACCGAGCCCCATTCGACCTTACAGAAGGAGA
GTCAGAACTCGTCTCTGGCTTCAACTAGAATTTATTCCTAACTATCTCCTTCCGAATTCGAGCATCATACCCCCGATTCCGATATGATCAACTTATACACCTATTATGAAAAAACTTCCTACCCC
TAACTCTAGCCTTATGTATATGACACGTATCCCTTCCCATCATAACTGCAAGTATTCCACCCCAAACATAAGAAATATGTCTGACAAAAGAGTTACTTTGATAGAGTAAATAATAGAGGTTTAAG
CCCTCTTATTTCTAGAGCTATAGGAATCGAACCCAATCCTAAGAATTCAAAAATCTTTGTGCTACCATATTACACCACGCTCTATAGTAAGGTCAGCTAAGTAAGCTATCGGGCCCATACCCCGA
AAATGTTGGTTTATACCCTTCCCGTACTAATTAAACCGCCTATTCTCATCATCATTATATCAACTGTAATCTCAGGGAAATTGTAAAGCTCTAACAATCTGAATCGGCTTTGAAATAAATATTCT
TATAAAAAAATTTAACCCCCGAGCTATAGAAGCAGCTACAAAATATTTCCTCACACAAGCAACCGCATCCATACTATTAATAATAGGAATTATCATCAAATAAACCCTCACCCCATAGCATCAAT
TATAATAACCACAGCCCTAGCAATAAAACTAGGTCTAGCCCCATTCCACTTCTGAGTACCCGAAGTAGCACAAGGAATTTCCCTATCCTCAAGTCTCATTCTATTAACATGACAAAAATACTTCT
AACAATAGCTATTACATCAGTTATCATCGGGGGCTGAGGAGGACTAAATCAAACTCAACTACGAAAAATCATAGCATATTCCTCAATCGCACACATATTCATACATAGCTCTTCTACCACAACAT
ATCCCTATCCCAAACATAAACAAAGCACCCTTGATCACCTCACTACTATTATCCCCACATACTAGCCATCACAGCACTACTCAATCTATACTTCTACATACGACTAACATACGCCACAGCACTTA
CTATATTTCCCTCAGCTAAAACATAAAAATAACACTTTCAACTATACTCTCCCTCTGCCCCCATAATATCCATCCTGGATTAGAAGTTTAGGTTAGATAGACCAAGAGCCTTCAAAGCCCTAAGC
AAGCCTCACTGGCTTAACTTCTGAACTCCTATAAGGACTGCAAGAATCTATCTCACATCAATTGATTGCAAATCAAACACTTTAATTAAGCTAAGCCCTTACTAGATTGGTGGGATCCAACCCCA
CGAAACTTTAGTTAACAGCTAAACACCCTAATCAACTGGCTTCAATCTACTTCTCCCGCCGTCTAGAAAAAAAAGGCGGGAGAAGCCCCGGCAGCGTCAAAGCTGCTTCTTTGAATTTGCAATTC
AACATGAATATTCACCACAGGACTTGGTAAAAAGAGGATTAAACCTCTGTTCTTAGATTTACAGTCTAATGCTTTGCTCAGCCATTTTACCTATGTTCATAAACCGTTGATTATTTTCAACTAAC
CACAAAGATATCGGAACTCTATTCAATAGTAGAAGCCGGGGCAGGAACCGGATGAACAGTTCTAGAGGTAATTTAGCACATGCAGGAGCATCGTAGACACCACAATCATTAACATAAAACCTCCT
GCTATATCCCAATACCAAACACCTTTATTTGTCTGATCAGTCTTAATCACGCTGTTCTATTCGTGTGAGCTCACCATATGTTTACTGTAGGCATGGATGTAGACACACGAGCATATTTTACATCA
GCTACTATAATTATCGCAATTATGTAGTAGCCCATTTCCACTATGTATTATCAATAGGAGCAGTTTTCGCTATTATAGGAGGCTTTGTACATTGATTTCCTTTATTTGAAGAACTGCCTACGTAT
CTTTAAAGTAAGAAAGGAAGGAATCGAACCTCCTAGGACTGGTTTCAAGCCAATATCATAACCATTACACTCTGAGCGGTCCATCACTAGGACTATAAATCAAACAACTTTAATAGGCACACGTC
CAGGGTTATACTATGGTCAATGCTCAGAAATCTGTGGCTCAAACCATAGTTTTATACCTATCGTCCTTGAATTAGTACCATTAACATATTTCGAGAAACCCCTGAAATCCAGAACCTAAATCTGC
AATTACACTAAAACAACCTAATCCTTGAGAAAAAAAATGAACGAAAATCTATTCGCCTCTTTCACTACCCCAACGATAATAGGCTTGCCCATTGTTATCTTAATCGTATTATTATCCTAGTCCTA
CTAACAATCCTTGAATTCGCAGTAGCCCTAATTCAAGCCTATGTCTTTACCCTGCTAGTAAGCTTATACTTACATGATCATATATGTACCAATGATGACGAGATGTTATTCGAGAAAGCACATTT
CAAGGCCACCACACACCCGTTGTTCAAAAAGGATTACGATATGGAATAATCCTATTTATTGATCTGAAGCTGAGCATTTTATCATTCAAGCTCCAGATTAGGAGGTTGCTGACCCACAGGCATTA
TTCCTCTAATCCTCTAGAAGTACCATTACTTAATACCTCTGTACTATTAGCTTCTGGAGTATCAATCACTTGAGCCCACCATAGCTTAATAGAAGGTAATCGAAAACACATCTTATACGACAACT
GAAATACCACTTTACATCAAACCATCATTTTGGATTTGAAGCCGCTGCTTGATATTGACATTTCGTAGATGTAGTGTGACTATTCCTTTACGTAATACTTTATTATCCTCACTCCTCGTTTTAAT
TGCATTTTGACTACCCCAATTAAATATTTACGCAGAAAAAGCAAGCCCCTATGAATGCGGATTTGACCCAATAGGATCTGCTCGTCTGCCTTTCTCTATAAATTTTTCTTACATTTCTACTATTT
GACCTAGAAATTGCACTACTATTACCCCTACCCTGAGCCTCACAAACAAATAACTTATAACAATACTTACTCTTTTAATTCGCTCTTGGCTGCAAGCCTAGCTTATGAATGAACCCAAAAAGGAC
TAGAATGATCAGAATATGATAATTATGCCTGTGAAGCAGCCCTGGTTAATATTATCCTCGAACTATAATTCTAGCACGAGGACTACAGACATCCTCCCTCTAATGGTAAATCACCCACCATATCA
AAAGCATCAAGCCATCCTTCACACGAGAAAACGCCCTAATAACCCTTCACCTGCTACCCCTTTTATTACTATCACTAAACCCCATAAATTCATGCTCCCATGTATGAAAACATGGCTTTTTCAAC
TTTTATAGGATAGAAGTAATCCATTGGCCTTAGGAGCCAAAAAATTGGTGCAACTCCAAATAAAAGTAATAAATTCATTCGCCTCCTTTATATTAACAGACCGCCAACAACCTATTCCAACTATT
TATCGGATGAGAAGGAGTGGGAATTATATCCTTTCTACTAATTGGCTGATGATATGGCCGAACTGACGCAAATACTGCCGCTCTACAGGCCATCTTATATAACCGTATCGATAGCCATAGATGTT
TCTTACTAACCTAAACGAATCAACCTTATCTCGCATTTCTGCACATCTGCACGCATGCATTCTTCAAAGCCATATTATTTATATGCTCAGGATCAATTATCCACAGCCTAAATGACGAGCAAGAT
ATCCGAAAAATAGGCGGACTTTATAAAGCTATACCATTTACCACCACCTCCCTAATTATCGGAAGCCTCGCACTCACAGGAATACCCTTCCTAACAGGCTTTTACTCCAAAGACCTAATTATCGA
GACCGCCAATACGTCGTATACCAACGCCTGAGCCCTATTAACTACTCTCATCGCCACATCCCTGACAGCTGCCTACAGCACTCGAATTATATTTTTTGCACTTCTAGGACAACCACGCTTCAACC
TTAATATGAGCCAAAAATCCGCATCAATACTACTAGACATAATCTGATTAAAAATACCTCCTCACTAAAAAACCCTGAATCTCCCGTATCATAAATTACTCAATCACCTGCACCATTAAATTTAA
ACACAATCTCAACCCATCCCCTTTAAAATATAGATTAAAAAAACCATTAACCCTAAAAAGGACCCCCCAAAGTTCAATACAATACCACAACCAACTCCACCAGCTACAATCAAACCAAACCCACC
ATAAATAGGTGAAGGCTTTGAAGAAAAACTCACAAAGCTAACCACAAAAATAGTACTTAAAATGAATACAATGTATGTCATCATTATTCTCACATGGAATCTAACCATGACTAATGATATGAAAA
ACCATCGTTGTATTTCAACTACAAGAATTAATGACCAACATCCGAAAATCCCACCCCTAATAAAATCATCAACGAATCTTTTATTGATCTTCCAACTCCACTATATCTCAGCATGATGAAACTTT
GGTTCCCTATTAGGAATCTGCCTAGTTCTACAAATCTTAACAGGCTTATTTCTAGCTATACACTACACATCAGACACAGCAACCGCCTTCTCATCAGTAACCCATATTTGCCGCGACGTCAACTA
CGGATGAATTATCCGATATATACATGCTAATGGAGCACTGATCTGGTAGAATGAATCTGAGGCGGCTTCTCAGTAGACAAGGCCACCTTGACACGGTTTTTCGCCTTTCACTTCATCCTTCCATT
CATCATCGCCGCTCTAGCAGCAGTCCACCTACTATTCCTTCATGAAACTGGGTCAAACAACCCCTCAGGTAAATCCCCACCCCATATCAAACCCGAATGGTATTTTCTATTCGCATACGCAATTC
TCCGATCCATCCCTAATAAATTAGGAGGAATCTCCTAAAGTGAAGAGTCTTTGTAGTATATAAAATACCTTGGTCTTGTAAACCAAAAAAGGAGAACATGATCCCTCCCTAAGACTTCAAGGAAG
```

AAGCAACTGCCCCACCACCAGCACCCAAAGCTGAAATTCTTTTTAAACTATTCCTTGCTAATACCAAAAATCAGCTCTCCAACATTCATAATTCATATATTGCATGA
>PLI-33_merged.sort.StEndRmdup_2_
TCCAAGTTAATGTAGCTTAAACCATAAAGCAAGGCACTGAAAATGCCTAGATGAGTTGTAATAACTCCATAAACATAAAGGTTTGGTCCTGGCCTTTCTATTAGTTATTAGTAGGATTACACATG
CAAGCCTCCGCATCCCGGTGAAAATACCCTCTATGTCATTCATGACCCAAAGGAGTAGACATCAAGCACACAACCACTGTAGCTCATGACGTCTTGCAAAGCCACACCCCCACGGGATACAGCAG
TGATAAAAATTAAGCTATGAATGAAAGTTCGACTAAGCCATACTAAACTTAAGGGTTGGTAAATTTCGTGCCAGCCACCGCGGTCATACGATTAACCCGAACTAATAGATCTACGGCGTAAAGCG
TGTTAAAGAAATAAGCCTCACTAGAGTTAAGTCTTAACTAACAGAAAGTAGTCTAATATCTCTAACTACACGATAGCTAAGATCCAAACTGGGATTAGATACCCCACTATGCTTAGCCCTAAACC
TAGATGACTTTACCCAACAAAGTTATCCGCCAGAGAACTACTAGCAACAGCTTAAAACTCAAAGGACTTGGCGGTGCTTTATATCCCTCTAGAGGAGCCTGTTCTATAATCGATAAACCCCGATA
TACCTCACCACTCCTTGCTAATCCAGTTTATATACCGCCATCTTCAGCAAACCCTTAAAAGGAATAAAAGTAAGCACAAGTATTTTTAACACAAAAAAGTTAGGTCAAGGTGTAACCTATGGAGT
GGGAAGTAATGGGCTACATTTTCTGATTATGAAATTAAGAACTAAAGGAGGATTTAGCAGTAAATTGAGAATAGAGTGCTCAATTGAATCGGGCCATGAAGCACGCACACACCGCCCGTCACCCT
CCTCAAGTGACAACCTATAAATAACATAATTATCCCATAAAATCACAAGAGGAGATAAGTCGTAACAAGGTAAGCATACTGGAAAGTGTGCTTGGATAACCAAGATGTAGCTTAAGTAAAGCATC
TGGCCTACACCCAGAAGATTTCATATTAAACTGAACATCCTGAGCTAAAACTAGCCCAAACAACTATAAACTCAACTTTCACTCAACAATAAATCAACCATTTAATTACTAAAATTAAAAGTATA
GGAGATAGAAATTTAACTTGGCGCCATAGAGATAGTACCGCAAGGGAAAGATGAAAGAAATATTTAAAGCACCACACAGCAAAGATTATCCCTTGTACCTTTTGCATAATGAATCAGCTAGAACG
CCCTAGCAAAGAGAACTTAAGCTAGACTCCCCGAAACCAGACGAGCTACCTGTGAACAATCTACATGGGATGAACTCATCTATGTCGCAAAATAGTGAGAAGATTTACAGGTAGAGGTGAAAAGC
CTAACGAGCCTGGTGATAGCTGGTTGCCCAGGATAGAATTTTAGTTCAACTTTAAACTTACCCGCAAATCTCCCTTAAAATTCTAATGTAAGTTTAAAATATAATCTAAAAAGGTACAGCTTTTT
AGAACTAGATACAGCCTTTTTTAGTGAGTAAGCATAATTATTAACCATAGTCGGCCTAAAAGCAGCCATCAATTAAGAAAGCGTTCAAGCTCAACAGTTAAAATATCTTAATATCAAAAGTAATT
ATATAATAGAAGCAATAATGCTGATATGAGTAACAAGAAATACTTCTCCTTGCATAAGCTTATATCAGAGCGGATAACCACTGATAGTTAACAACAAGATATGAAAAACCTACCTATAAATTCAA
TATCAAGTTAATTGTTACCCAACACAGGCATGCAATTAAGGAAAGATTAAAAGAAGTAAAAGGAACTCGGCAAACACAAGCCCCGCCTGTTTACCAAAAACATCACCTCTAGCATTCCTAGTATT
AGAGGCACTGCCTGCCCAGTGACATTAGTTAAACGGCCGCGGTATCCTGACCGTGCAAAGGTAGCATAATCACTTGTTCCCTAAATAGGGACTCGTATGAATGGCCACACGAGGGCTTTACTGTC
TCTTACTTCCAATCCGTGAAATTGACCTTCCCGTGAAGAGGCGGGAATGTGACAATAAGACGAGAAGACCCTATGGAGCTTTAATTAACTAACTCAGAGAGATTACTTTAACATACGATAACAAA
CCTCTATATGAGTTAGCAATTGGGTTGGGGTGACCTCGGAGAACAAAAAAACCTCCGAGTGATTTTAATCTAGACTAACCAGTCAAAAGTATTACATCACTTATTGATCCAAAAACTTGATCAAC
GGAACAAGTTACCCTAGGGATAACAGCGCAATCCTATTTTAGAGTCCATATCGACAATAGGGTTTACGACCTCGATGTTGGATCAGGACATCCCGATGGTGCAGCAGCTATCAAAGGTTCGTTTG
TTCAACGATTAAAGTCCTACGTGATCTGAGTTCAGACCGGAGTAATCCAGGTCGGTTTCTATCTATTAAACAATTTCTCCCAGTACGAAAGGACAAGAGAAATAGGGCCTACCTTACCAAGACGC
CCTCAACTGTAGATGTACGGGTTAGTTAGGGTGGCAGAGCCCGGTAATTGCATAAAACTTAAACTTTTACAATCAGAGGTTCAATTCCTCTCCCTAACAATATGTTCATAATTAATATTTTATCA
CTAATCGTTCCAATCCTTCTCGCCGTAGCCTTCCTAACACTAGTTGAACGTAAAGTCTTAGGTTACATACAACTCCGCAAGGGACCAAACATTGTAGGACCATACGGTCTGCTCCAACCCATCGC
AGACGCCATTAAATTATTTACCAAAGAACCCCTGCGACCCCTAACATCCTCCATATCAATATTCATCATAGCACCAATCCTAGCCCTTCACTGCTCTAACCATATGAATCATAAACTTCATACTA
GCCATATAAGTTTAGCCGTATATTCCATTTTATGCCTCAAACTCCAAATATGCCTTAATTGGAGCTCTACGAGCCGTAGCCCAAACAATCTCATATGAAGTCACACTAGCTATCATTCTCCTATC
CGTGCTCTAATTACCACCCAAGAATATATATGGCTAATCTTTCCCGCATGACCCCTAGCCATAATATGATTTATCTCAACTCTAGCAGAAACCAACCGAGCCCCATTCGACCTTACAGAAGGAGA
GTCAGAACTCGTCTCTGGCTTCAACTAGAATTTATTCCTAACTATCTCCTTCCGAATTCGAGCATCATACCCCCGATTCCGATATGATCAACTTATACACCTATTATGAAAAAACTTCCTACCCC
TAACTCTAGCCCTATGTATATGACACGTATCCCTTCCCATCATAACTGCAAGTATTCCACCCCAAACATAAGAAATATGTCTGACAAAAGAGTTACTTTGATAGAGTAAATAATAGAGGTTTAAG
CCCTCTTATTTCTAGAGCTATAGGAATCGAACCCAATCCTAAGAATTCAAAAATCTTTGTGCTACCATATTACACCACGCTCTATAGTAAGGTCAGCTAAGTAAGCTATCGGGCCCATACCCCGA
AAATGTTGGTTTATACCCTTCCCGTACTAATTAAACCGCCTATTCTCATCATCATTATATCAACTGTAATCTCAGGGAAATTGTAAAGCTCTAACAATCTGAATCGGCTTTGAAATAAACATTCT
TATAAAAAAATTTAACCCCCGAGCTATAGAAGCAGCTACAAAATATTTCCTCACACAAGCAACCGCATCCATACTATTAATAATAGGAATTATCATCAAATAAACCCTCACCCCATAGCATCAAT
TATAATAACCACAGCCCTAGCAATAAAACTAGGTCTAGCCCCATTCCACTTCTGAGTACCCGAAGTAGCACAAGGAATTTCCCTATCCTCAAGTCTCATTCTATTAACATGACAAAAATACTTCT
AACAATAGCTATTACATCAGTTATCATCGGGGGCTGAGGAGGACTAAATCAAACTCAACTACGAAAAATCATAGCATATTCCTCAATCGCACACATATTCATACATAGCTCTTCTACCACAACAT
ATCCCTATCCCAAACATAAACAAAGCACCCTTAATCACCTCACTATTATTATCCCCACATACTAGCCATCACAGCACTACTCAATCTATACTTCTACATACGACTAACATACGCCACAGCACTTA
CTATATTTCCCTCAGCTAAAACATAAAAATAACACTTTCAACTATACTCTCCCTCTGCCCCCATAATATCCATCCTGGATTAGAAGTTTAGGTTAGATAGACCAAGAGCCTTCAAAGCCCTAAGC
AAGCCTCACTGGCTTAACTTCTGAACTCCCATAAGGACTGCAAGAATCTATCTCACATCAATTGATTGCAAATCAAACACTTTAATTAAGCTAAGCCCTTACTAGATTGGTGGGATCCAACCCCA
CGAAACTTTAGTTAACAGCTAAACACCCTAATCAACTGGCTTCAATCTACTTCTCCCGCCGTCTAGAAAAAAAAGGCGGGAGAAGCCCCGGCAGCGTCAAAGCTGCTTCTTTGAATTTGCAATTC
AACATGAATATTCACCACAGGACTTGGTAAAAAGAGGATTAAACCTCTGTTCTTAGATTTACAGTCTAATGCTTTGCTCAGCCATTTTACCTATGTTCATAAACCGTTGATTATTTTCAACTAAC
CACAAAGATATCGGAACTCTATTCAATAGTAGAAGCCGGGGCAGGAACCGGATGAACAGTTCTAGAGGTAATTTAGCACATGCAGGAGCATCGTAGACACCACAATCATTAACATAAAACCTCCT
GCTATATCCCAATACCAAACACCTTTATTTGTCTGATCAGTCTTAATCACGCTGTTCTATTCGTGTGAGCTCACCATATGTTTACTGTAGGCATGGATGTAGACACACGAGCATATTTTACATCA
GCTACTATAATTATCGCAATTATGTAGTAGCCCATTTCCACTATGTATTATCAATAGGAGCAGTTTTCGCTATTATAGGAGGCTTTGTACATTGATTTCCTTTATTTGAAGAACTGCCTACGTAT
CTTTAAAGTAAGAAAGGAAGGAATCGAACCTCCTAGGACTGGTTTCAAGCCAATATCATAACCATTACACTCTGAGCGGTCCATCACTAGGACTATAAATCAAACAACTTTAATAGGCACACGTC
CAGGGTTATACTATGGTCAATGCTCAGAAATCTGTGGCTCAAACCATAGTTTTATACCTATTGTCCTTGAATTAGTACCATTAACATATTTCGAGAAACCCCTGAAATCCAGAACCTAAATCTGC
AATTACACTAAAACAACCTAATCCTTGAGAAAAAAAATGAACGAAAATCTATTCGCCTCTTTCACTACCCCAACGATAATAGGCTTGCCCATTGTTATCTTAATCGTATTATTATCCTAGTCCTA
CTAACAATCCTTGAATTCGCAGTAGCCCTAATTCAAGCCTATGTCTTTACCCTGCTAGTAAGCCTATACTTACATGATCATATATGTACCAATGATGACGAGATGTTATTCGAGAAAGCACGTTT
CAAGGCCACCACACACCCGTTGTTCAAAAAGGATTACGATATGGAATAATCCTATTTATTGATCTGAAGCTGAGCATTTTATCATTCAAGCTCCAGATTAGGAGGTTGCTGACCCACAGGCATTA
TTCCTCTAATCCTCTAGAAGTACCATTACTTAATACCTCTGTACTATTAGCTTCTGGAGTATCAATCACTTGAGCCCACCATAGCTTAATAGAAGGTAATCGAAAACACATCTTATACGACAACT
GAAATACCACTTTACATCAAACCATCATTTTGGATTTGAAGCCGCTGCTTGATATTGACATTTCGTAGATGTAGTGTGACTATTCCTTTACGTAATACTTTATTATCCTCACTCCTCGTTTTAAT
TGCATTTTGACTACCACAATTAAATATTTACGCAGAAAAAGCAAGCCCCTATGAATGCGGATTTGACCCAATAGGATCTGCTCGTCTGCCTTTCTCTATAAATTTTTCTTACATTTCTACTATTT
GACCTAGAAATTGCACTACTATTACCCCTACCCTGAGCCTCACAAACAAATAACTTATAACAATACTTACTCTTTTAATTCGCTCTTGGCTGCAAGCCTAGCTTATGAATGAACCCAAAAAGGGC
TAGAATGATCAGAATATGATAATTATGCCTGTGAAGCAGCCCTGGTTAATATTATCCTCGAACTATAATTCTAGCACGAGGACTACAGACATCCTCCCTCTAATGGTAAATCACCCACCATATCA
AAAGCATCAAGCCATCCTTCACACGAGAAAACGCCCTAATAACCCTTCACCTGCTACCCCTTTTATTACTATCACTAAACCCCATAAATTCATGCTCCCATGTATGAAAACATGGCTTTTTCAAC
TTTTATAGGATAGAAGTAATCCATTGGCCTTAGGAGCCAAAAAATTGGTGCAACTCCAAATAAAAGTAATAAATTCATTCGCCTCCTTTATATTAACAGACCGCCAACAACCTATTCCAACTATT
TATCGGATGAGAAGGAGTGGGAATTATATCCTTTCTACTAATTGGCTGATGATATGGCCGAACTGACGCAAATACTGCCGCTCTACAGGCCATCTTATATAATCGTATCGATAGCCATAGATGTT
TCTTACTAACCTAAACGAATCAACCTTATCTCGCATTTCTGCACATCTGCACGCATGCATTCTTCAAAGCCATATTATTTATATGCTCAGGATCAATTATCCACAGCCTAAATGACGAGCAAGAT
ATCCGAAAAATAGGCGGACTTTATAAATCTATACCATTTACCACCACCTCCCTAATTATCGGAAGCCTCGCACTCACAGGAATACCCTTCCTAACAGGCTTTTACTCCAAAGACCTAATTATCGA
GACCGCCAATACGTCGTATACCAACGCCTGAGCCCTATTAACTACTCTCATCGCCACATCCCTGACAGCTGCCTACAGCACTCGAATTATATTTTTTGCACTTCTAGGACAACCACGCTTCAACC
TTAATATGAGCCAAAAATCCGCATCAATACTACTAGACATAATCTGATTAAAAATACCTCCTCACTAAAAAACCCTGAATCTCCCGTATCATAAATTACTCAATCACCCGCACCATTAAATTTAA
ACACAATCTCAACCCATCCCCTTTAAAATATAGATTAAAAAAACCATTAACCCTAAAAAGGACCCCCCAAAGTTCAATACAATACCACAACCAACTCCACCAGCTACAATCAAACCAAACCCACC
ATAAATAGGTGAAGGCTTTGAAGAAAAACTCACAAAGCTAACCACAAAAATAGTACTTAAAATGAATACAATGTATGTCATCATTATTCTCACATGGAATCTAACCATGACTAATGATATGAAAA
ACCATCGTTGTATTTCAACTACAAGAATTAATGACCAACATCCGAAAATCCCACCCCTAATAAAATCATCAACGAATCCTTTATTGATCTTCCAACTCCACTATATCTCAGCATGATGAAACTTT
GGTTCCCTATTAGGAATCTGCCTAGTTCTACAAATCCTAACAGGCTTATTTCTAGCCATACACTACACATCAGACACAGCAACCGCCTTCTCATCAGTAACCCATATTTGCCGCGACGTCAACTA
CGGATGAATTATCCGATATATACATGCTAATGGAGCACTGATCTGGTAGAATGAATCTGAGGCGGCTTCTCAGTAGACAAGGCCACCTTGACACGGTTTTTCGCCTTTCACTTCATCCTTCCATT
CATCATCGCCGCCCTAGCAGCAGTCCACCTACTATTCCTTCATGAAACTGGATCAAACAACCCCTCAGGTAAATCCCCACCCCATATCAAACCCGAATGGTATTTTCTATTCGCATACGCAATTC
TCCGATCCATCCCTAATAAATTAGGAGGAATCTCCTAAAATGAAGAGTCTTTGTAGTATATAAAATACCTTGGTCTTGTAAACCAAAAAAGGAGAACATGATCCCTCCCTAAGACTTCAAGGAAG
AAGCAACTGCCCCACCACCAGCACCCAAAGCTGAAATTCTTTTTAAACTATTCCTTGCTAATACCAAAAATCAGCTCTCCAACATTCATAATTCATATATTGCATGA
>PLI-39.sort.StEndRmdup_3x-90p
TCCAAGTTAATGTAGCTTAAACCATAAAGCAAGGCACTGAAAATGCCTAGATGAGTTGTAATAACTCCATAAACATAAAGGTTTGGTCCTGGCCTTTCTATTAGTTATTAGTAGGATTACACATG
CAAGCCTCCGCATCCCGGTGAAAATACCCTCTATGTCATTCATGACCCAAAGGAGTAGACATCAAGCACACAACCACTGTAGCTCATGACGTCTTGCAAAGCCACACCCCCACGGGATACAGCAG
TGATAAAAATTAAGCTATGAATGAAAGTTCGACTAAGCCATACTAAACTTAAGGGTTGGTAAATTTCGTGCCAGCCACCGCGGTCATACGATTAACCCGAACTAATAGATCTACGGCGTAAAGCG
TGTTAAAGAAATAAGCCTCACTAGAGTTAAGTCTTAACTAACAGAAAGTAGTCTAATATCTCTAACTACACGATAGCTAAGATCCAAACTGGGATTAGATACCCCACTATGCTTAGCCCTAAACC
TAGATGACTTCACCCAACAAAGTTATCCGCCAGAGAACTACTAGCAACAGCTTAAAACTCAAAGGACTTGGCGGTGCTTTATATCCCTCTAGAGGAGCCTGTTCTATAATCGATAAACCCCGATA
TACCTCACCACTCCTTGCTAATCCAGTTTATATACCGCCATCTTCAGCAAACCCTTAAAAGGAATAAAAGTAAGCACAAGTATTTTTAACACAAAAAAGTTAGGTCAAGGTGTAACCTATGGAGT
GGGAAGTAATGGGCTACATTTTCTGATTATGAAATTAAGAACTAAAGGAGGATTTAGCAGTAAATTGAGAATAGAGTGCTCAATTGAATCGGGCCATGAAGCACGCACACACCGCCCGTCACCCT
CCTCAAGTGACAACCTATAAATAACATAATTATCCCATAAAATCACAAGAGGAGATAAGTCGTAACAAGGTAAGCATACTGGAAAGTGTGCTTGGATAACCAAGATGTAGCTTAAGTAAAGCATC
TGGCCTACACCCAGAAGATTTCATATTAAACTGAACATCCTGAGCTAAAACTAGCCCAAACAACTATAAACTCAACTTTCACTCAACAATAAATCAACCATTTAATTACTAAAATTAAAAGTATA
GGAGATAGAAATTTAACTTGGCGCCATAGAGATAGTACCGCAAGGGAAAGATGAAAGAAATATTTAAAGCACCACACAGCAAAGATTATCCCTTGTACCTTTTGCATAATGAATCAGCTAGAACG
CCCTAGCAAAGAGAACTTAAGCTAGACTCCCCGAAACCAGACGAGCTACCTGTGAACAATCTACATGGGATGAACTCATCTATGTCGCAAAATAGTGAGAAGATTTACAGGTAGAGGTGAAAAGC
CTAACGAGCCTGGTGATAGCTGGTTGCCCAGGATAGAATTTTAGTTCAACTTTAAACTTACCCGCAAATCTCCCTTAAAATTCTAATGTAAGTTTAAAATATAATCTAAAAAGGTACAGCTTTTT
AGAACTAGATACAGCCTTTTTTAGTGAGTAAGCATAATTATTAACCATAGTCGGCCTAAAAGCAGCCATCAATTAAGAAAGCGTTCAAGCTCAACAGTTAAAATATCTTAATATCAAAAGTAATT
ATATAATAGAAGCAATAATGCTGATATGAGTAACAAGAAATACTTCTCCTTGCATAAGCTTATATCAGAGCGGATAACCACTGATAGTTAACAACAAGATATGAAAAACCCACCTATAAATTCAA
TATCAAGTTAATTGTTACCCAACACAGGCATGCAATCAAGGAAAGATTAAAAGAAGTAAAAGGAACTCGGCAAACACAAGCCCCGCCTGTTTACCAAAAACATCACCTCTAGCATTTCTAGTATT
AGAGGCACTGCCTGCCCAGTGACATTAGTTAAACGGCCGCGGTATCCTGACCGTGCAAAGGTAGCATAATCACTTGTTCCCTAAATAGGGACTCGTATGAATGGCCACACGAGGGCTTTACTGTC
TCTTACTTCCAATCCGTGAAATTGACCTTCCCGTGAAGAGGCGGGAATGTGACAATAAGACGAGAAGACCCTATGGAGCTTTAATTAACTAACTCAGAGAGATTACTTTAACATACGATAACAAA
CCTCTATATGAGTTAGCAATTGGGTTGGGGTGACCTCGGAGAACAAAAAAACCTCCGAGTGATTTTAATCTAGACTAACCAGTCAAAAGTATTACATCACTTATTGATCCAAAAACTTGATCAAC
GGAACAAGTTACCCTAGGGATAACAGCGCAATCCTATTTTAGAGTCCATATCGACAATAGGGTTTACGACCTCGATGTTGGATCAGGACATCCCGATGGTGCAGCAGCTATCAAAGGTTCGTTTG
TTCAACGATTAAAGTCCTACGTGATCTGAGTTCAGACCGGAGTAATCCAGGTCGGTTTCTATCTATTAAACAATTTCTCCCAGTACGAAAGGACAAGAGAAATAGGGCCTACCTTACCAAGACGC
CCTCAACTGTAGATGTACGGGTTAGTTAGGGTGGCAGAGCCCGGTAATTGCATAAAACTTAAACTTTTACAATCAGAGGTTCAATTCCTCTCCCTAACAATATGTTCATAATTAATATTTTATCA

CTAATCGTTCCAATCCTTCTCGCCGTAGCCTTCCTAACACTAGTTGAACGTAAAGTCTTAGGTTACATACAACTCCGCAAGGGACCAAACATTGTAGGACCATACGGTCTGCTCCAACCCATTGC
AGACGCCATTAAATTATTTACCAAAGAACCCCTGCGACCCCTAACATCCTCCATATCAATATTCATCATAGCACCAATCCTAGCCCTTCACTGCCCTAACCATATGAATCATAAACTTCATACTA
GCCATATAAGTTTAGCCGTATATTCCATTTTATGCCTCAAACTCCAAATATGCCTTAATTGGAGCTCTACGAGCCGTAGCCCAAACAATCTCATATGAAGTCACACTAGCTATCATTCTCCTATC
CGTGCTCTAATTACCACCCAAGAATATATATGGCTAATCTTTCCCGCATGACCCCTAGCCATAATATGATTTATCTCAACTCTAGCAGAAACCAACCGAGCCCCATTCGACCTTACAGAAGGAGA
GTCAGAACTCGTCTCTGGCTTCAACTAGAATTTATTCCTAACTATCTCCTTCCGAATTCGAGCATCATACCCCCGATTCCGATATGATCAACTTATACACCTATTATGAAAAAACTTCCTACCCC
TAACTCTAGCCCTATGTATATGACACGTATCCCTTCCCATCATAACTGCAAGTATTCCACCCCAAACATAAGAAATATGTCTGACAAAAGAGTTACTTTGATAGAGTAAATAATAGAGGTTTAAG
CCCTCTTATTTCTAGAGCTATAGGAATCGAACCCAATCCTAAGAATTCAAAAATCTTTGTGCTACCATATTACACCACGCTCTATAGTAAGGTCAGCTAAGTAAGCTATCGGGCCCATACCCCGA
AAATGTTGGTTTATACCCTTCCCGTACTAATTAAACCGCCTATTCTCATCATCATTATATCAACTGTAATCTCAGGGAAATTGTAAAGCTCTAACAATCTGAATCGGCTTTGAAATAAACATTCT
CATAAAAAAATTTAACCCCCGAGCTATAGAAGCAGCTACAAAATATTTCCTCACACAAGCAACCGCATCCATACTATTAATAATAGGAATTATCATCAAATAAACCCTCACCCCATAGCATCAAT
TATAATAACCACAGCCCTAGCAATAAAACTAGGTCTAGCCCCATTCCACTTCTGAGTACCCGAAGTAGCACAAGGAATTTCCCTATCCTCAAGTCTCATTCTATTAACATGACAAAAATACTTCT
AACAATAGCCATTACATCAGTTATCATCGGGGGCTGAGGAGGACTAAATCAAACTCAACTACGAAAAATCATAGCATATTCCTCAATCGCACACATATTCATACATAGCTCTTCTACCACAACAT
ATCCCTATCCCAAACATAAACAAAGCACCCTTGATCACCTCACTACTATTATCCCCACATACTAGCCATCACAGCACTACTCAATCTATACTTCTACATACGACTAACATACGCCACAGCACTTA
CTATATTTCCCTCAGCTAAAACATAAAAATAACACTTTCAACTATACTCTTCCTCTGCCCCCATAATATCCATCCTGGATTAGAAGTTTAGGTTAGATAGACCAAGAGCCTTCAAAGCCCTAAGC
AAGCCTCACTGGCTTAACTTCTGAACTCCCATAAGGACTGCAAGAATCTATCTCACATCAATTGATTGCAAATCAAACACTTTAATTAAGCTAAGCCCTTACTAGATTGGTGGGATCCAACCCCA
CGAAACTTTAGTTAACAGCTAAACACCCTAATCAACTGGCTTCAATCTACTTCTCCCGCCGTCTAGAAAAAAAAAGGCGGGAGAAGCCCCGGCAGCGTCAAAGCTGCTTCTTTGAATTTGCAATTC
AACATGAATATTCACCACAGGACTTGGTAAAAAGAGGATTAAACCTCTGTTCTTAGATTTACAGTCTAATGCTTTGCTCAGCCATTTTACCTATGTTCATAAACCGTTGATTATTTTCAACTAAC
CACAAAGATATCGGAACTCTATTCAATAGTAGAAGCCGGGGCAGGAACCGGATGAACAGTTCTAGAGGTAATTTAGCACATGCAGGAGCATCGTAGACACCACAATCATTAACATAAAACCCCCT
GCTATATCCCAATACCAAACACCTTTATTTGTCTGATCAGTCTTAATCACGCTGTTCTATTCGTGTGAGCTCACCATATGTTTACTGTAGGCATGGATGTAGACACACGAGCATATTTTACATCA
GCTACTATAATTATCGCAATTATGTAGTAGCCCATTTCCACTATGTATTATCAATAGGAGCAGTTTTCGCTATTATAGGAGGCTTTGTACATTGATTTCCTTTATTTGAAGAACTGCCTACGTAT
CTTTAAAGTAAGAAAGGAAGGAATCGAACCTCCTAGGACTGGTTTCAAGCCAATATCATAACCATTACACTCTGAGCGGTCCATCACTAGGACTATAAATCAAACAACTTTAATAGGCACACGTC
CAGGGTTATACTATGGTCAATGCTCAGAAATCTGTGGCTCAAACCATAGTTTTATACCTATCGTCCTTGAATTAGTACCATTAACATATTTCGAGAAACCCCTGAAATCCAGAACCTAAATCTGC
AATTATACTAAAACAACCTAATCCTTGAGAAAAAAAATGAACGAAAATCTATTCGCCTCTTTCACTACCCCAACGATAATAGGCTTGCCCATTGTTATCTTAATCGTATTATTATCCTAGTCCTA
CTAACAATCCTTGAATTCGCAGTAGCCCTAATTCAAGCCTATGTCTTTACCCTGCTAGTAAGCCTATACTTACATGATCATATATGTACCAATGATGACGAGATGTTATTCGAGAAAGCACGTTT
CAAGGCCACCACACACCCGTTGTTCAAAAAGGATTACGATATGGAATAATCCTATTTATTGATCTGAAGCTGAGCATTTTATCATTCAAGCTCCAGATTAGGAGGTTGCTGACCCACAGGCATTA
TTCCTCTAATCCTCTAGAAGTACCATTACTTAATACCTCTGTACTATTAGCTTCTGGAGTATCAATCACTTGAGCCCACCATAGCTTAATAGAAGGTAATCGAAAACACATCTTATACGACAACT
GAAATACCACTTTACATCAAACCATCATTTTGGATTTGAAGCCGCTGCTTGATATTGACATTTCGTAGATGTAGTGTGACTATTCCTTTACGTAATACTTTATTATCCTCACTCCTCGTTTTAAT
TGCATTTTGACTACCCCAATTAAATATTTACGCAGAAAAAGCAAGCCCCTATGAATGCGGATTTGACCCAATAGGATCTGCTCGTCTGCCTTTCTCTATAAATTTTTCTTACATTTCTACTATTT
GACCTAGAAATTGCACTACTATTACCCCTACCCTGAGCCTCACAAACAAATAACTTATAACAATACTTACTCTTTTAATTCACTCTTGGCTGCAAGCCTAGCTTATGAATGAACCCAAAAAGGAC
TAGAATGATCAGAATATGATAATTATGCCTGTGAAGCAGCCCTGGTTAATATTATCATCGAACTATAATTCTAGCACGAGGACTACAGACATCCTCCCTCTAATGGTAAATCACCCACCATATCA
AAAGCATCAAGCCATCCTTCACACGAGAAAACGCCCTAATAACCCTTCACCTGCTACCCCTTTTATTACTATCACTAAACCCCATAAATTCATGCTCCCATGTATGAAAACATGGCTTTTTCAAC
TTTTATAGGATAGAAGTAATCCATTGGCCTTAGGAGCCAAAAATTTGGTGCAACTCCAAATAAAAGTAATAAATTCATTCGCCTCCTTTATATTAACAGACCGCCAACAACCTATTCCAACTATT
TATCGGATGAGAAGGAGTGGGAATTATATCCTTTCTACTAATTGGCTGATGATACGGCCGAACTGACGCAAATACTGCCGCTCTACAGGCCATCTTATATAACCGTATCGATAGCCATAGATGTT
TCTTACTAACCTAAACGAATCAACCTTATCTCGCATTTCTGCACATCTGCACGCATGCATTCTTCAAAGCCATATTATTTATATGCTCAGGATCAATTATTCACAGCCTAAATGACGAGCAAGAT
ATCCGAAAAATAGGCGGACTTTATAAATCTATACCATTTACCACCACCTCCCTAATTATCGGAAGCCTCGCACTCACAGGAATACCATTCCTAACAGGCTTTTACTCCAAAGACCTAATTATCGA
GACCGCCAATACGTCGTATACCAACGCCTGAGCCCTATTAACTACTCTCATCGCCACATCCCTGACAGCTGCCTACAGCACTCGAATTATATTTTTTGCACTTCTAGGACAACCACGCTTCAACC
TTAATATGAGCCAAAAATCCGCATCAATACTACTAGACATAATCTGATTAAAAATACCTCCTCACTAAAAAACCCTGAATCTCCCGTATCATAAATTACTCAATCACCCGCACCATTAAATTTAA
ACACAATCTCAACCCATCCCCTTTAAAATATAGATTAAAAAGACCATTAACCCTAAAAAGGACCCCCCAAAGTTCAATACAATACCACAACCAACTCCACCAGCTACAATCAAACCAAACCCACC
ATAAATAGGTGAAGGCTTTGAAGAAAAACTCACAAAGCTAACCACAAAAATAGTACTTAAAATGAATACAATGTATGTCATCATTATTCTCACATGGAATCTAACCATGACTAATGATATGAAAA
ACCATCGTTGTATTTCAACTACAAGAATTAATGACCAACATCCGAAAATCCCACCCCTAATAAAATTATCAACGAATCCTTTATTGATCTTCCAACTCCACTATATCTCAGCATGATGAAACTTT
GGTTCCCTATTAGGAATCTGCCTAGTTCTACAAATCTTAACAGGCTTATTTCTAGCCATACACTACACATCAGACACAGCAACCGCCTTCTCATCAGTAACCCATATTTGCCGCGACGTCAACTA
CGGATGAATTATCCGATATATACATGCTAATGGAGCACTGATCTGGTAGAATGAATCTGAGGCGGCTTCTCAGTAGACAAGGCCACCTTGACACGGTTTTTCGCCTTTCACTTCATCCTTCCATT
CATCATCGCCGCTCTAGCAGCAGTCCACCTACTATTCCTTCATGAAACTGGATCAAACAACCCCTCAGGTAAATCCCCACCCCATATCAAACCCGAATGGTATTTTCTATTCGCATACGCAATTC
TCCGATCCATCCCTAATAAACTAGGAGGAATCTCCTAAAGTGAAGAGTCTTTGTAGTATATAAAATACCTTGGTCTTGTAAACCAAAAAAGGAGAACATGATCCCTCCCTAAGACTTCAAGGAAG
AAGCAACTGCCCCACCACCAGCACCCAAAGCTGAAATTCTTTTTAAACTATTCCTTGCTAATACCAAAAATCAGCTCTCCAACATTCATAATTCATATATTGCATGA
>PLI-28.sort.StEndRmdup_3x-90p
TCCAAGTTAATGTAGCTTAAACCATAAAGCAAGGCACTGAAAATGCCTAGATGAGTTGTAATAACTCCATAAACATAAAGGTTTGGTCCTGGCCTTTCTATTAGTTATTAGTAGGATTACACATG
CAAGCCTCCGCATCCCGGTGAAAATACCCTCTATGTCATTCATGACCCAAAGGAGTAGACATCAAGCACACAACCACTGTAGCTCATGACGTCTTGCAAAGCCACACCCCCACGGGATACAGCAG
TGATAAAAATTAAGCTATGAATGAAAGTTCGACTAAGCCATACTAAACTTAAGGGTTGGTAAATTTCGTGCCAGCCACCGCGGTCATACGATTAACCCGAACTAATAGATCTACGGCGTAAAGCG
TGTTAAAGAAATAAGCCTCACTAGAGTTAAGTCTTAACTAACAGAAAGTAGTCTAATATCTCTAACTACACGATAGCTAAGATCCAAACTGGGATTAGATACCCCACTATGCTTAGCCCTAAACC
TAGATGACTTCACCCAACAAAGTTATCCGCCAGAGAACTACTAGCAACAGCTTAAAACTCAAAGGACTTGGCGGTGCTTTATATCCCTCTAGAGGAGCCTGTTCTATAATCGATAAACCCCGATA
TACCTCACCACTCCTTGCTAATCCAGTTTATATACCGCCATCTTCAGCAAACCCTTAAAAGGAATAAAAGTAAGCACAAGTATTTTTAACACAAAAAAGTTAGGTCAAGGTGTAACCTATGGAGT
GGGAAGTAATGGGCTACATTTTCTGATTATGAAATTAAGAACTAAAGGAGGATTTAGCAGTAAATTGAGAATAGAGTGCTCAATTGAATCGGGCCATGAAGCACGCACACACCGCCCGTCACCCT
CCTCAAGTGACAACCTATAAATAACATAATTATCCCATAAAATCACAAGAGGAGATAAGTCGTAACAAGGTAAGCATACTGGAAAGTGTGCTTGGATAACCAAGATGTAGCTTAAGTAAAGCATC
TGGCCTACACCCAGAAGATTTCATATTAAACTGAACATCCTGAGCTAAAACTAGCCCAAACAACTATAAACTCAACTTTCACTCAACAATAAATCAACCATTTAATTACTAAAATTAAAAGTATA
GGAGATAGAAATTTAACTTGGCGCCATAGAGATAGTACCGCAAGGGAAAGATGAAAGAAATATTTAAAGCACCACACAGCAAAGATTATCCCTTGTACCTTTTGCATAATGAATCAGCTAGAACG
CCCTAGCAAAGAGAACTTAAGCTAGACTCCCCGAAACCAGACGAGCTACCTGTGAACAATCTACATGGGATGAACTCATCTATGTCGCAAAATAGTGAGAAGATTTACAGGTAGAGGTGAAAAGC
CTAACGAGCCTGGTGATAGCTGGTTGCCCAGGATAGAATTTTAGTTCAACTTTAAACTTACCCGCAAATCTCCCTTAAAATTCTAATGTAAGTTTAAAATATAATCTAAAAAGGTACAGCTTTTT
AGAACTAGATACAGCCTTTTTTAGTGAGTAAGCATAATTATTAACCATAGTCGGCCTAAAAGCAGCCATCAATTAAGAAAGCGTTCAAGCTCAACAGTTAAAATATCTTAATATCAAAAGTAATT
ATATAATAGAAGCAATAATGCTGATATGAGTAACAAGAAATACTTCTCCTTGCATAAGCTTATATCAGAGCGGATAACCACTGATAGTTAACAACAAGATATGAAAAACCCACCTATAAATTCAA
TATCAAGTTAATTGTTACCCAACACAGGCATGCAATCAAGGAAAGATTAAAAGAAGTAAAAGGAACTCGGCAAACACAAGCCCCGCCTGTTTACCAAAAACATCACCTCTAGCATTTCTAGTATT
AGAGGCACTGCCTGCCCAGTGACATTAGTTAAACGGCCGCGGTATCCTGACCGTGCAAAGGTAGCATAATCACTTGTTCCCTAAATAGGGACTCGTATGAATGGCCACACGAGGGCTTTACTGTC
TCTTACTTCCAATCCGTGAAATTGACCTTCCCGTGAAGAGGCGGGAATGTGACAATAAGACGAGAAGACCCTATGGAGCTTTAATTAACTAACTCAGAGAGATTACTTTAACATACGATAACAAA
CCTCTATATGAGTTAGCAATTGGGTTGGGGTGACCTCGGAGAACAAAAAAACCTCCGAGTGATTTTAATCTAGACTAACCAGTCAAAAGTATTACATCACTTATTGATCCAAAAACTTGATCAAC
GGAACAAGTTACCCTAGGGATAACAGCGCAATCCTATTTTAGAGTCCATATCGACAATAGGGTTTACGACCTCGATGTTGGATCAGGACATCCCGATGGTGCAGCAGCTATCAAAGGTTCGTTTG
TTCAACGATTAAAGTCCTACGTGATCTGAGTTCAGACCGGAGTAATCCAGGTCGGTTTCTATCTATTAAACAATTTCTCCCAGTACGAAAGGACAAGAGAAATAGGGCCTACCTTACCAAGACGC
CCTCAACTGTAGATGTACGGGTTAGTTAGGGTGGCAGAGCCCGGTAATTGCATAAAACTTAAACTTTTACAATCAGAGGTTCAATTCCTCTCCCTAACAATATGTTCATAATTAATATTTTATCA
CTAATCGTTCCAATCCTTCTCGCCGTAGCCTTCCTAACACTAGTTGAACGTAAAGTCTTAGGTTACATACAACTCCGCAAGGGACCAAACATTGTAGGACCATACGGTCTGCTCCAACCCATTGC
AGACGCCATTAAATTATTTACCAAAGAACCCCTGCGACCCCTAACATCCTCCATATCAATATTCATCATAGCACCAATCCTAGCCCTTCACTGCCCTAACCATATGAATCATAAACTTCATACTA
GCCATATAAGTTTAGCCGTATATTCCATTTTATGCCTCAAACTCCAAATATGCCTTAATTGGAGCTCTACGAGCCGTAGCCCAAACAATCTCATATGAAGTCACACTAGCTATCATTCTCCTATC
CGTGCTCTAATTACCACCCAAGAATATATATGGCTAATCTTTCCCGCATGACCCCTAGCCATAATATGATTTATCTCAACTCTAGCAGAAACCAACCGAGCCCCATTCGACCTTACAGAAGGAGA
GTCAGAACTCGTCTCTGGCTTCAACTAGAATTTATTCCTAACTATCTCCTTCCGAATTCGAGCATCATACCCCCGATTCCGATATGATCAACTTATACACCTATTATGAAAAAACTTCCTACCCC
TAACTCTAGCCCTATGTATATGACACGTATCCCTTCCCATCATAACTGCAAGTATTCCACCCCAAACATAAGAAATATGTCTGACAAAAGAGTTACTTTGATAGAGTAAATAATAGAGGTTTAAG
CCCTCTTATTTCTAGAGCTATAGGAATCGAACCCAATCCTAAGAATTCAAAAATCTTTGTGCTACCATATTACACCACGCTCTATAGTAAGGTCAGCTAAGTAAGCTATCGGGCCCATACCCCGA
AAATGTTGGTTTATACCCTTCCCGTACTAATTAAACCGCCTATTCTCATCATCATTATATCAACTGTAATCTCAGGGAAATTGTAAAGCTCTAACAATCTGAATCGGCTTTGAAATAAACATTCT
TATAAAAAAATTTAACCCCCGAGCTATAGAAGCAGCTACAAAATATTTCCTCACACAAGCAACCGCATCCATACTATTAATAATAGGAATTATCATCAAATAAACCCTCACCCCATAGCATCAAT
TATAATAACCACAGCCCTAGCAATAAAACTAGGTCTAGCCCCATTCCACTTCTGAGTACCCGAAGTAGCACAAGGAATTTCCCTATCCTCAAGTCTCATTCTATTAACATGACAAAAATACTTCT
AACAATAGCCATTACATCAGTTATCATCGGGGGCTGAGGAGGACTAAATCAAACTCAACTACGAAAAATCATAGCATATTCCTCAATCGCACACATATTCATACATAGCTCTTCTACCACAACAT
ATCCCTATCCCAAACATAAACAAAGCACCCTTGATCACCTCACTACTATTATCCCCACATACTAGCCATCACAGCACTACTCAATCTATACTTCTACATACGACTAACATACGCCACAGCACTTA
CTATATTTCCCTCAGCTAAAACATAAAAATAACACTTTCAACTATACTCTTCCTCTGCCCCCATAATATCCATCCTGGATTAGAAGTTTAGGTTAGATAGACCAAGAGCCTTCAAAGCCCTAAGC
AAGCCTCACTGGCTTAACTTCTGAACTCCCATAAGGACTGCAAGAATCTATCTCACATCAATTGATTGCAAATCAAACACTTTAATTAAGCTAAGCCCTTACTAGATTGGTGGGATCCAACCCCA
CGAAACTTTAGTTAACAGCTAAACACCCTAATCAACTGGCTTCAATCTACTTCTCCCGCCGTCTAGAAAAAAAAAGGCGGGAGAAGCCCCGGCAGCGTCAAAGCTGCTTCTTTGAATTTGCAATTC
AACATGAATATTCACCACAGGACTTGGTAAAAAGAGGATTAAACCTCTGTTCTTAGATTTACAGTCTAATGCTTTGCTCAGCCATTTTACCTATGTTCATAAACCGTTGATTATTTTCAACTAAC
CACAAAGATATCGGAACTCTATTCAATAGTAGAAGCCGGGGCAGGAACCGGATGAACAGTTCTAGAGGTAATTTAGCACATGCAGGAGCATCGTAGACACCACAATCATTAACATAAAACCCCCT
GCTATATCCCAATACCAAACACCTTTATTTGTCTGATCAGTCTTAATCACGCTGTTCTATTCGTGTGAGCTCACCATATGTTTACTGTAGGCATGGATGTAGACACACGAGCATATTTTACATCA
GCTACTATAATTATCGCAATTATGTAGTAGCCCATTTCCACTATGTATTATCAATAGGAGCAGTTTTCGCTATTATAGGAGGCTTTGTACATTGATTTCCTTTATTTGAAGAACTGCCTACGTAT
CTTTAAAGTAAGAAAGGAAGGAATCGAACCTCCTAGGACTGGTTTCAAGCCAATATCATAACCATTACACTCTGAGCGGTCCATCACTAGGACTATAAATCAAACAACTTTAATAGGCACACGTC
CAGGGTTATACTATGGTCAATGCTCAGAAATCTGTGGCTCAAACCATAGTTTTATACCTATCGTCCTTGAATTAGTACCATTAACATATTTCGAGAAACCCCTGAAATCCAGAACCTAAATCTGC
AATTATACTAAAACAACCTAATCCTTGAGAAAAAAAATGAACGAAAATCTATTCACCTCTTTCACTACCCCAACGATAATAGGCTTGCCCATTGTTATCTTAATCGTATTATTATCCTAGTCCTA
CTAACAATCCTTGAATTCGCAGTAGCCCTAATTCAAGCCTATGTCTTTACCCTGCTAGTAAGCCTATACTTACATGATCATATATGTACCAATGATGACGAGATGTTATTCGAGAAAGCACGTTT

CAAGGCCACCACACACCCGTTGTTCAAAAAGGATTACGATATGGAATAATCCTATTTATTGATCTGAAGCTGAGCATTTTATCATTCAAGCTCCAGATTAGGAGGTTGCTGACCCACAGGCATTA
TTCCTCTAATCCTCTAGAAGTACCATTACTTAATACCTCTGTACTATTAGCTTCTGGAGTATCAATCACTTGAGCCCACCATAGCTTAATAGAAGGTAATCGAAAACACATCTTATACGACAACT
GAAATACCACTTTACATCAAACCATCATTTTGGATTTGAAGCCGCTGCTTGATATTGACATTTCGTAGATGTAGTGTGACTATTCCTTTACGTAATACTTTATTATCCTCACTCCTCGTTTTAAT
TGCATTTTGACTACCCCAATTAAATATTTACGCAGAAAAAGCAAGCCCCTATGAATGCGGATTTGACCCAATAGGATCTGCTCGTCTGCCTTTCTCTATAAATTTTTCTTACATTTCTACTATTT
GACCTAGAAATTGCACTACTATTACCCCTACCCTGAGCCTCACAAACAAATAACTTATAACAATACTTACTCTTTTAATTCACTCTTGGCTGCAAGCCTAGCTTATGAATGAACCCAAAAAGGAC
TAGAATGATCAGAATATGATAATTATGCCTGTGAAGCAGCCCTGGTTAATATTATCATCGAACTATAATTCTAGCACGAGGACTACAGACATCCTCCCTCTAATGGTAAATCACCCACCATATCA
AAAGCATCAAGCCATCCTTCACACGAGAAAACGCCCTAATAACCCTTCACCTGCTACCCCTTTTATTACTATCACTAAACCCCATAAATTCATGCTCCCATGTATGAAAACATGGCTTTTTCAAC
TTTTATAGGATAGAAGTAATCCATTGGCCTTAGGAGCCAAAAATTTGGTGCAACTCCAAATAAAAGTAATAAATTCATTCGCCTCCTTTATATTAACAGACCGCCAACAACCTATTCCAACTATT
TATCGGATGAGAAGGAGTGGGAATTATATCCTTTCTACTAATTGGCTGATGATACGGCCGAACTGACGCAAATACTGCCGCTCTACAGGCCATCTTATATAACCGTATCGATAGCCATAGATGTT
TCTTACTAACCTAAACGAATCAACCTTATCTCGCATTTCTGCACATCTGCACGCATGCATTCTTCAAAGCCATATTATTTATATGCTCAGGATCAATTATTCACAGCCTAAATGACGAGCAAGAT
ATCCGAAAAATAGGCGGACTTTATAAATCTATACCATTTACCACCACCTCCCTAATTATCGGAAGCCTCGCACTCACAGGAATACCATTCCTAACAGGCTTTTACTCCAAAGACCTAATTATCGA
GACCGCCAATACGTCGTATACCAACGCCTGAGCCCTATTAACTACTCTCATCGCCACATCCCTGACAGCTGCCTACAGCACTCGAATTATATTTTTTGCACTTCTAGGACAACCACGCTTCAACC
TTAATATGAGCCAAAAATCCGCATCAATACTACTAGACATAATCTGATTAAAAATACCTCCTCACTAAAAAACCCTGAATCTCCCGTATCATAAATTACTCAATCACCCGCACCATTAAATTTAA
ACACAATCTCAACCCATCCCCTTTAAAATATAGATTAAAAAGACCATTAACCCTAAAAAGGACCCCCCAAAGTTCAATACAATACCACAACCAACTCCACCAGCTACAATCAAACCAAACCCACC
ATAAATAGGTGAAGGCTTTGAAGAAAAACTCACAAAGCTAACCACAAAAATAGTACTTAAAATGAATACAATGTATGTCATCATTATTCTCACATGGAATCTAACCATGACTAATGATATGAAAA
ACCATCGTTGTATTTCAACTACAAGAATTAATGACCAACATCCGAAAATCCCACCCCTAATAAAATTATTAACGAATCCTTTATTGATCTTCCAACTCCACTATATCTCAGCATGATGAAACTTT
GGTTCCCTATTAGGAATCTGCCTAGTTCTACAAATCTTAACAGGCTTATTTCTAGCCATACACTACACATCAGACACAGCAACCGCCTTCTCATCAGTAACCCATATTTGCCGCGACGTCAACTA
CGGATGAATTATCCGATATATACATGCTAATGGAGCACTGATCTGGTAGAATGAATCTGAGGCGGCTTCTCAGTAGACAAGGCCACCTTGACACGGTTTTTCGCCTTTCACTTCATCCTTCCATT
CATCATCGCCGCTCTAGCAGCAGTCCACCTACTATTCCTTCATGAAACTGGATCAAACAACCCCTCAGGTAAATCCCCACCCCATATCAAACCCGAATGGTATTTTCTATTCGCATACGCAATTC
TCCGATCCATCCCTAATAAACTAGGAGGAATCTCCTAAAGTGAAGAGTCTTTGTAGTATATAAAATACCTTGGTCTTGTAAACCAAAAAAGGAGAACATGATCCCTCCCTAAGACTTCAAGGAAG
AAGCAACTGCCCCACCACCAGCACCCAAAGCTGAAATTCTTTTTAAACTATTCCTTGCTAATACCAAAAATCAGCTCTCCAACATTCATAATTCATATATTGCATGA
>PLI-8_merged.sort.StEndRmdup_2_3
TCCAAGTTAATGTAGCTTAAACCATAAAGCAAGGCACTGAAAATGCCTAGATGAGTTGTAATAACTCCATAGACATAAAGGTTTGGTCCTGGCCTTTCTATTAGTTATTAGTAGGATTACACATG
CAAGCCTCCGCATCCCGGTGAAAATACCCTCTATGTCATTCATGACCCAAAGGAGTAGACATCAAGCACACAACCACTGTAGCTCATGACGTCTTGCAAAGCCACACCCCCACGGGATACAGCAG
TGATAAAAATTAAGCTATGAATGAAAGTTCGACTAAGCTATACTAAACTTAAGGGTTGGTAAATTTCGTGCCAGCCACCGCGGTCATACGATTAACCCGAACTAATAGATCTACGGCGTAAAGCG
TGTTAAAGAAATAAGCCTCACTAGAGTTAAGTCTTAACTAACAGAAAGTAGTCTAATATCTCTAACTACACGATAGCTAAGATCCAAACTGGGATTAGATACCCCACTATGCTTAGCCCTAAACC
TAGATGACTTTACCCAACAAAGTTATCCGCCAGAGAACTACTAGCAACAGCTTAAAACTCAAAGGACTTGGCGGTGCTTTATATCCCTCTAGAGGAGCCTGTTCTATAATCGATAAACCCCGATA
TACCTCACCACTCCTTGCTAATCCAGTTTATATACCGCCATCTTCAGCAAACCCTTAAAAGGAATAAAAGTAAGCACAAGTATTTTTAACACAAAAAAGTTAGGTCAAGGTGTAACCTATGGAGT
GGGAAGTAATGGGCTACATTTTCTGATTATGAAATTAAGAACTAAAGGAGGATTTAGCAGTAAATTGAGAATAGAGTGCTCAATTGAATCGGGCCATGAAGCACGCACACACCGCCCGTCACCCT
CCTCAAGTGACAACCTATAAATAACATAATTATCCCATAAAATCACAAGAGGAGATAAGTCGTAACAAGGTAAGCATACTGGAAAGTGTGCTTGGATAACCAAGATGTAGCTTAAGTAAAGCATC
TGGCCTACACCCAGAAGATTTCATATTAAACTGAACATCCTGAGCTAAAACTAGCCCAAACAACTATAAACTCAACTTTCACTCAACAATAAATCAACCATTTAATTACTAAAATTAAAAGTATA
GGAGATAGAAATTTAACTTGGCGCCATAGAGATAGTACCGCAAGGGAAAGATGAAAGAAATATTTAAAGCACCACACAGCAAAGATTATCCCTTGTACCTTTTGCATAATGAATCAGCTAGAACG
CCCTAGCAAAGAGAACTTAAGCTAGACCCCCCGAAACCAGACGAGCTACCTGTGAACAATCTACATGGGATGAACTCATCTATGTCGCAAAATAGTGAGAAGATTTACAGGTAGAGGTGAAAAGC
CTAACGAGCCTGGTGATAGCTGGTTGCCCAGGATAGAATTTTAGTTCAACTTTAAACTTACCCGCAAATCTCCCTTAAAATTCTAATGTAAGTTTAAAATATAATCTAAAAAGGTACAGCTTTTT
AGAACTAGATACAGCCTTTTTTAGTGAGTAAGCATAATTATTAACCATAGTCGGCCTAAAAGCAGCCATCAATTAAGAAAGCGTTCAAGCTCAACAGTTAAAATATCTTAATATCAAAAGTAATT
ATATAATAGAAGCAATAATGCTGATATGAGTAACAAGAAATATTTCTCCTTGCATAAGCTTATATCAGAGCGGATAACCACTGATAGTTAACAACAAGATATGAAAAACCTACCTATGAATTCAA
TATCAAGTTAATTGTTACCCAACACAGGCATGCAATCAAGGAAAGATTAAAAGAAGTAAAAGGAACTCGGCAAACACAAGCCCCGCCTGTTTACCAAAAACATCACCTCTAGCATTTCTAGTATT
AGAGGCACTGCCTGCCCAGTGACATTAGTTAAACGGCCGCGGTATCCTGACCGTGCAAAGGTAGCATAATCACTTGTTCCCTAAATAGGGACTCGTATGAATGGCCACACGAGGGCTTTACTGTC
TCTTACTTCCAATCCGTGAAATTGACCTTCCCGTGAAGAGGCGGGAATGTGACAATAAGACGAGAAGACCCTATGGAGCTTTAATTAACTAACTCAGAGAGATTACTTTAACATACGATAACAAA
CCTCTATATGAGTTAGCAATTGGGTTGGGGTGACCTCGGAGAACAAAAAAACCTCCGAGTGATTTTAATCTAGACTAACCAGTCAAAAGTATTACATCACTTATTGATCCAAAAACTTGATCAAC
GGAACAAGTTACCCTAGGGATAACAGCGCAATCCTATTTTAGAGTCCATATCGACAATAGGGTTTACGACCTCGATGTTGGATCAGGACATCCCGATGGTGCAGCAGCTATCAAAGGTTCGTTTG
TTCAACGATTAAAGTCCTACGTGATCTGAGTTCAGACCGGAGTAATCCAGGTCGGTTTCTATCTATTAAACAATTTCTCCCAGTACGAAAGGACAAGAGAAATAGGGCCTACCTTACCAAGACGC
CCTCAACTGTAGATGTACGGGTTAGTTAGGGTGGCAGAGCCCGGTAATTGCATAAAACTTAAACTTTTACAATCAGAGGTTCAATTCCTCTCCCTAACAATATGTTCATAATTAATATTTTATCA
CTAATCGTTCCAATCCTTCTCGCCGTAGCCTTCCTAACACTAGTTGAACGCAAAGTCTTAGGTTACATACAACTCCGCAAGGGACCAAACATTGTAGGACCATACGGTCTGCTCCAACCCATCGC
AGACGCCATTAAATTATTTACCAAAGAACCCCTGCGACCCCTAACATCCTCCATATCAATATTCATCATAGCACCAATCCTAGCCCTTCACTGCTCTAACCATATGAATCATAAACTTCATACTA
GCCATATAAGTTTAGCCGTATATTCCATTTTATGCCTCAAACTCCAAATATGCCTTAATTGGAGCTCTACGAGCCGTAGCCCAAACAATCTCATATGAAGTCACACTAGCTATCATTCTCCTATC
CGTGCTCTAATTACCACCCAAGAATATATATGGCTAATCTTTCCCGCATGACCCCTAGCCATAATATGATTTATCTCAACTCTAGCAGAAACCAACCGAGCCCCATTCGACCTTACAGAAGGAGA
GTCAGAACTCGTCTCTGGCTTCAACTAGAATTTATTCCTAACTATCTCCTTCCGAATTCGAGCATCATACCCCCGATTCCGATATGATCAACTTATACACCTATTATGAAAAAACTTCCTACCCC
TAACTCTAGCCTTATGTATATGACACGTATCCCTTCCCATCATAACTGCAAGTATTCCACCCCAAACATAAGAAATATGTCTGACAAAAGAGTTACTTTGATAGAGTAAATAATAGAGGTTTAAG
CCCTCTTATTTCTAGAGCTATAGGAATCGAACCCAATCCTAAGAATTCAAAAATCTTTGTGCTACCATATTACACCACGCTCTATAGTAAGGTCAGCTAAGTAAGCTATCGGGCCCATACCCCGA
AAATGTTGGTTTATACCCTTCCCGTACTAATTAAACCGCCTATTCTCATCATCATTATATCAACTGTAATCTCAGGGAAATTGTAAAGCTCTAACAATCTGAATCGGCTTTGAAATAAACATTCT
TATAAAAAAATTTAACCCCCGAGCTATAGAAGCAGCTACAAAATATTTCCTCACACAAGCAACCGCATCCATACTATTAATAATAGGAATTATCATCAAATAAACCCTCACCCCATAGCATCAAT
TATAATAACCACAGCCCTAGCAATAAAACTAGGTCTAGCCCCATTCCACTTCTGAGTACCCGAAGTAGCACAAGGAATTTCCCTATCCTCAAGTCTCATTCTATTAACATGACAAAAATACTTCT
AACAATAGCTATTACATCAGTTATCATCGGGGGCTGAGGAGGACTAAATCAAACTCAACTACGAAAAATCATAGCATATTCCTCAATCGCACACATATTCATACATAGCTCTTCTACCACAACAT
ATCCCTATCCCAAACATAAACAAAGCACCCTTGATCACCTCACTACTATTATCCCCACATACTAGCCATCACAGCACTACTCAATCTATACTTCTACATACGACTAACATACGCCACAGCACTTA
CTATATTTCCCTCAGCTAAAACATAAAAATAACACTTTCAACTATACTCTCCCTCTGCCCCCATAATATCCATCCTGGATTAGAAGTTTAGGTTAGATAGACCAAGAGCCTTCAAAGCCCTAAGC
AAGCCTCACTGGCTTAACTTCTGAACTCCTATAAGGACTGCAAGAATCTATCTCACATCAATTGATTGCAAATCAAACACTTTAATTAAGCTAAGCCCTTACTAGATTGGTGGGATCCAACCCCA
CGAAACTTTAGTTAACAGCTAAACACCCTAATCAACTGGCTTCAATCTACTTCTCCCGCCGTCTAGAAAAAAAAGGCGGGAGAAGCCCCGGCAGCGTCAAAGCTGCTTCTTTGAATTTGCAATTC
AACATGAATATTCACCACAGGACTTGGTAAAAAGAGGATTAAACCTCTGTTCTTAGATTTACAGTCTAATGCTTTGCTCAGCCATTTTACCTATGTTCATAAACCGTTGATTATTTTCAACTAAC
CACAAAGATATCGGAACTCTATTCAATAGTAGAAGCCGGGGCAGGAACCGGATGAACAGTTCTAGAGGTAATTTAGCACATGCAGGAGCATCGTAGACACCACAATCATTAACATAAAACCTCCT
GCTATATCCCAATACCAAACACCTTTATTTGTCTGATCAGTCTTAATCACGCTGTTCTATTCGTGTGAGCTCACCATATGTTTACTGTAGGCATGGATGTAGACACACGAGCATATTTTACATCA
GCTACTATAATTATCGCAATTATGTAGTAGCCCATTTCCACTATGTATTATCAATAGGAGCAGTTTTCGCTATTATAGGAGGCTTTGTACATTGATTTCCTTTATTTGAAGAACTGCCTACGTAT
CTTTAAAGTAAGAAAGGAAGGAATCGAACCTCCTAGGACTGGTTTCAAGCCAATATCATAACCATTACACTCTGAGCGGTCCATCACTAGGACTATAAATCAAACAACTTTAATAGGCACACGTC
CAGGGTTATACTATGGTCAATGCTCAGAAATCTGTGGCTCAAACCATAGTTTTATACCTATCGTCCTTGAATTAGTACCATTAACATATTTCGAGAAACCCCTGAAATCCAGAACCTAAATCTGC
AATTACACTAAAACAACCTAATCCTTGAGAAAAAAAATGAACGAAAATCTATTCGCCTCTTTCACTACCCCAACGATAATAGGCTTGCCCATTGTTATCTTAATCGTATTATTATCCTAGTCCTA
CTAACAATCCTTGAATTCGCAGTAGCCCTAATTCAAGCCTATGTCTTTACCCTGCTAGTAAGCTTATACTTACATGATCATATATGTACCAATGATGACGAGATGTTATTCGAGAAAGCACATTT
CAAGGCCACCACACACCCGTTGTTCAAAAAGGATTACGATATGGAATAATCCTATTTATTGATCTGAAGCTGAGCATTTTATCATTCAAGCTCCAGATTAGGAGGTTGCTGACCCACAGGCATTA
TTCCTCTAATCCTCTAGAAGTACCATTACTTAATACCTCTGTACTATTAGCTTCTGGAGTATCAATCACTTGAGCCCACCATAGCTTAATAGAAGGTAATCGAAAACACATCTTATACGACAACT
GAAATACCACTTTACATCAAACCATCATTTTGGATTTGAAGCCGCTGCTTGATATTGACATTTCGTAGATGTAGTGTGACTATTCCTTTACGTAATACTTTATTATCCTCACTCCTCGTTTTAAT
TGCATTTTGACTACCCCAATTAAATATTTACGCAGAAAAAGCAAGCCCCTATGAATGCGGATTTGACCCAATAGGATCTGCTCGTCTGCCTTTCTCTATAAATTTTTCTTACATTTCTACTATTT
GACCTAGAAATTGCACTACTATTACCCCTACCCTGAGCCTCACAAACAAATAACTTATAACAATACTTACTCTTTTAATTCGCTCTTGGCTGCAAGCCTAGCTTATGAATGAACCCAAAAAGGAC
TAGAATGATCAGAATATGATAATTATGCCTGTGAAGCAGCCCTGGTTAATATTATCCTCGAACTATAATTCTAGCACGAGGACTACAGACATCCTCCCTCTAATGGTAAATCACCCACCATATCA
AAAGCATCAAGCCATCCTTCACACGAGAAAACGCCCTAATAACCCTTCACCTGCTACCCCTTTTATTACTATCACTAAACCCCATAAATTCATGCTCCCATGTATGAAAACATGGCTTTTTCAAC
TTTTATAGGATAGAAGTAATCCATTGGCCTTAGGAGCCAAAAAATTGGTGCAACTCCAAATAAAAGTAATAAATTCATTCGCCTCCTTTATATTAACAGACCGCCAACAACCTATTCCAACTATT
TATCGGATGAGAAGGAGTGGGAATTATATCCTTTCTACTAATTGGCTGATGATATGGCCGAACTGACGCAAATACTGCCGCTCTACAGGCCATCTTATATAACCGTATCGATAGCCATAGATGTT
TCTTACTAACCTAAACGAATCAACCTTATCTCGCATTTCTGCACATCTGCACGCATGCATTCTTCAAAGCCATATTATTTATATGCTCAGGATCAATTATCCACAGCCTAAATGACGAGCAAGAT
ATCCGAAAAATAGGCGGACTTTATAAAGCTATACCATTTACCACCACCTCCCTAATTATCGGAAGCCTCGCACTCACAGGAATACCCTTCCTAACAGGCTTTTACTCCAAAGACCTAATTATCGA
GACCGCCAATACGTCGTATACCAACGCCTGAGCCCTATTAACTACTCTCATCGCCACATCCCTGACAGCTGCCTACAGCACTCGAATTATATTTTTTGCACTTCTAGGACAACCACGCTTCAACC
TTAATATGAGCCAAAAATCCGCATCAATACTACTAGACATAATCTGATTAAAAATACCTCCTCACTAAAAAACCCTGAATCTCCCGTATCATAAATTACTCAATCACCTGCACCATTAAATTTAA
14ACACAATCTCAACCCATCCCCTTTAAAATATAGATTAAAAAAACCATTAACCCTAAAAAGGACCCCCCAAAGTTCAATACAATACCACAACCAACTCCACCAGCTACAATCAAACCAAACCCA
CCATAAATAGGTGAAGGCTTTGAAGAAAAACTCACAAAGCTAACCACAAAAATAGTACTTAAAATGAATACAATGTATGTCATCATTATTCTCACATGGAATCTAACCATGACTAATGATATGAA
AAACCATCGTTGTATTTCAACTACAAGAATTAATGACCAACATCCGAAAATCCCACCCCTAATAAAATCATCAACGAATCTTTTATTGATCTTCCAACTCCACTATATCTCAGCATGATGAAACT
TTGGTTCCCTATTAGGAATCTGCCTAGTTCTACAAATCTTAACAGGCTTATTTCTAGCTATACACTACACATCAGACACAGCAACCGCCTTCTCATCAGTAACCCATATTTGCCGCGACGTCAAC
TACGGATGAATTATCCGATATATACATGCTAATGGAGCACTGATCTGGTAGAATGAATCTGAGGCGGCTTCTCAGTAGACAAGGCCACCTTGACACGGTTTTTCGCCTTTCACTTCATCCTTCCA
TTCATCATCGCCGCTCTAGCAGCAGTCCACCTACTATTCCTTCATGAAACTGGGTCAAACAACCCCTCAGGTAAATCCCCACCCCATATCAAACCCGAATGGTATTTTCTATTCGCATACGCAAT
TCTCCGATCCATCCCTAATAAATTAGGAGGAATCTCCTAAAGTGAAGAGTCTTTGTAGTATATAAAATACCTTGGTCTTGTAAACCAAAAAAGGAGAACATGATCCCTCCCTAAGACTTCAAGGA
AGAAGCAACTGCCCCACCACCAGCACCCAAAGCTGAAATTCTTTTTAAACTATTCCTTGCTAATACCAAAAATCAGCTCTCCAACATTCATAATTCATATATTGCATGA

Supporting References

1. Eizirik E, Murphy WJ, Koepfli K-P, Johnson WE, Dragoo JW, Wayne RK, et al. Pattern and timing of diversification of the mammalian order Carnivora inferred from multiple nuclear gene sequences. Mol Phylogenet Evol. 2010;56: 49–63. doi:10.1016/j.ympev.2010.01.033
2. McKenna MC, Bell SK. Classification of Mammals: Above the Species Level. Columbia University Press; 1997.
3. Werdelin L, Lewis ME. Plio Pleistocene Carnivora of eastern Africa: species richness and turnover patterns. Zool J Linn Soc. 2005;144: 121–144. doi:10.1111/j.1096-3642.2005.00165.x
4. Van Valkenburgh B, Grady F, Kurtén B. The Plio-Pleistocene cheetah-like cat Miracinonyx inexpectatus of North America. J Vertebr Paleontol. 1990;10: 434–454. doi:10.1080/02724634.1990.10011827
5. Turner A, Antón M. The Big Cats and Their Fossil Relatives: An Illustrated Guide to Their Evolution and Natural History. Columbia University Press; 1997.
6. Lanfear R, Calcott B, Ho SYW, Guindon S. PartitionFinder: Combined Selection of Partitioning Schemes and Substitution Models for Phylogenetic Analyses. Mol Biol Evol. 2012;29: 1695–1701. doi:10.1093/molbev/mss020
7. Drummond AJ, Suchard MA, Xie D, Rambaut A. Bayesian Phylogenetics with BEAUti and the BEAST 1.7. Mol Biol Evol. 2012;29: 1969–1973. doi:10.1093/molbev/mss075
8. Stamatakis A. RAxML Version 8: A tool for Phylogenetic Analysis and Post-Analysis of Large Phylogenies. Bioinformatics. 2014; btu033. doi:10.1093/bioinformatics/btu033
9. Miller MA, Pfeiffer W, Schwartz T. The CIPRES Science Gateway: A Community Resource for Phylogenetic Analyses. Proceedings of the 2011 TeraGrid Conference: Extreme Digital Discovery. New York, NY, USA: ACM; 2011. pp. 41:1–41:8. doi:10.1145/2016741.2016785
10. Paijmans JLA, Barnett R, Gilbert MTP, Zepeda-Mendoza ML, Reumer JWF, Vos J de, et al. Evolutionary History of Saber-Toothed Cats Based on Ancient Mitogenomics. Curr Biol. 2017;27: 3330-3336.e5. doi:10.1016/j.cub.2017.09.033
